# Associations of sociodemographic, health, and work-related factors with early long-term economic inactivity in older workers: Findings from the Health and Employment After Fifty study

**DOI:** 10.1371/journal.pone.0359865

**Published:** 2026-10-06

**Authors:** Stefania D’Angelo, Georgia Ntani, Ira Madan, David Coggon

**Affiliations:** 1 University of Southampton, MRC Lifecourse Epidemiology Centre, Southampton, United Kingdom; 2 Occupational Health Service, Guy’s and St Thomas’ NHS Foundation Trust, London, United Kingdom; 3 King’s College London School of Population Health and Environmental Sciences, London, England, United Kingdom; Iran University of Medical Sciences, IRAN, ISLAMIC REPUBLIC OF

## Abstract

**Objectives:**

To explore determinants of early long-term economic inactivity (ELTEI) among older workers in England.

**Methods:**

We analysed data from the Health and Employment After Fifty (HEAF) study, which prospectively followed a population-based sample of individuals in England who were aged 50−64 years at recruitment in 2013−14. ELTEI was defined as being out of paid work continuously for 12 months or longer before state pension age. Risk factors for first onset of ELTEI were examined by discrete-time Cox regression, taking as time intervals the periods between successive annual questionnaires. Associations were summarised by hazard ratios (HRs).

**Results:**

During follow-up of 3,450 participants for up to five years (a total of 11,561 12-month intervals), 682 (19.8%) entered ELTEI. In adjusted models, the strongest risk factors reflected financial security: low contribution to family income (HR 2.06, 95%CI 1.30, 3.25 men; 1.66 95%CI 1.25, 2.19 women), expectation of receiving a private/employer pension (HR 2.14, 95%CI 1.27, 3.77 men), anticipated high retirement income (HR 2.27, 95%CI 1.50, 3.44 men; 1.39 95%CI 1.04, 1.85 women) and having no mortgage or rent commitment (HR 1.75, 95%CI 1.09, 2.83 men; 1.62 95%CI 1.02, 2.56 women). Job dissatisfaction (HR 1.83, 95%CI 1.24, 2.64 men; 1.40 95%CI 0.99, 1.99 women) was associated with increased risk, as was absence of high physical work demands, especially among women (HR 0.68, 95%CI 0.48, 0.97 women). The only significant health-related risk factor was report of slow walking speed (HR 3.17, 95%CI 1.84, 5.33 men; 1.77 95%CI 1.18, 2.69 women).

**Conclusions:**

In contrast to high annual sickness absence, ELTEI is more common among individuals with higher education and less physically demanding jobs and seems to be driven largely by considerations of personal finance and affordability.

## Introduction

Like many European countries, the UK is experiencing rapid population ageing, and its Government has implemented legislation to raise the age at which people become eligible to receive a state pension [1,2]. This limits a burden on the public exchequer, and by encouraging people to remain in paid work to an older age [3], it has potential to increase national productivity. Moreover, continued labour market participation may be beneficial for individuals by contributing to better health, wellbeing, and financial security in later life [4].

However, anticipated benefits of the policy will be reduced if older workers have high levels of sickness absence because of health problems associated with ageing, or if, although not yet receiving a state pension, substantial numbers leave employment before reaching their state pension age (SPA). We recently reported findings from the Health and Employment after Fifty (HEAF) study which pointed to disabling musculoskeletal pain, depression, high body mass index, and physically demanding work as independent determinants of high annual sickness absence in a population-based cohort of older people [5]. We here describe an analysis of the same cohort to explore risk factors for long-term exit from paid work at least 12 months before SPA.

Previous studies looking at determinants of early retirement or exit from employment have identified a range of influences, including aspects of health [6–10] and personal financial circumstances [8–10]. In addition, a study of non-disability early retirement in the Netherlands found higher risk among people who felt less appreciated at work [10]. As a guide to future policy, it would be helpful to have further information on the relative importance of such risk factors.

## Materials and methods

### The HEAF study

The HEAF study is a longitudinal investigation that was set up to explore the inter-relationship of health and work in late middle age. Its methods have previously been described in detail [11]. In brief, during 2012 the Clinical Practice Research Database (CPRD) invited all contributing general practices in England to support the study. Those that volunteered were identified to the research team and were included as recruitment centres. In total, 24 general practices contributed to the sampling frame, with geographical spread across the South, Midlands and North of England. During January 2013 to June 2014, each practice mailed an invitation with a baseline questionnaire and consent form to all registered patients aged 50–64 years, regardless of their employment status, but excluding any with terminal illness or recent bereavement (N = 35,359). Approximately a quarter (N = 8,134) consented and returned a baseline questionnaire giving information about demographic characteristics; work status; job characteristics; social and financial circumstances; retirement plans; and mental and physical health.

### Follow-up

At each of six annual follow-ups, participants reported any changes in their employment. If they had left the job held at the time of the previous questionnaire, they provided the leaving date, and indicated whether the departure was at least partly for health reasons. If they had subsequently entered a new job, they detailed its characteristics and the start date. The follow-up questionnaire also collected updated information on health status and social circumstances. In some instances, either the date of leaving a job or the date of starting a new one were missing. Where possible we applied a rule-based single imputation as follows: 1) where there was a missing leave date and no subsequent new job, the leave date was imputed as the midpoint between the dates of completing the current and previous questionnaires; 2) where there was a missing leave date but the date of starting a subsequent job was known, the leave date was imputed as the midpoint between the date of completing the previous questionnaire and the date of starting the new job; 3) where there was a job change and the date of leaving the first job was known but that of starting the new job was missing, the start date was imputed as the midpoint between the date of leaving the first job and that of completing the current questionnaire. No imputation was undertaken when both dates (leave and start job date) were missing.

### Outcome

The outcome analysed in this paper was first onset during follow-up of early long-term economic inactivity (ELTEI), which we defined as being continuously out of paid work for 12 months or longer before SPA. Within the cohort, SPA varied from 65 to 67 years in men and from 63 to 67 years in women, according to date of birth.

### Independent variables

Potential determinants of ELTEI were ascertained from the baseline and follow-up questionnaires and fell into five main categories: demographic characteristics; indicators of likely personal financial security when no longer working; time demands outside paid work (e.g., caring responsibilities); aspects of the individual’s job that might make continuing employment more or less attractive/necessary; and measures of ill-health which could make it more difficult to remain in paid work. All had been identified at the time when the study was being set up as plausible determinants of exit from paid work and are described fully in S1 Table. Demographic variables included age at baseline, highest educational attainment, area-level deprivation, general practice region, and marital status. Financial factors included the proportion of household income contributed by the participant, housing tenure, perceived financial difficulties, expectation of receiving a pension, and anticipated changes in total income following retirement. We also considered involvement in caring responsibilities and other unpaid work. Work-related risk factors included employment status, shift work, physical demands of work, difficult workplace relationships and/or job-related worries, perception of job as rewarding, having friends at work, and overall job satisfaction. Finally, a range of health-related variables were examined, including depressive symptoms, disabling musculoskeletal pain, somatising tendency, sleep problems, walking speed, body mass index (BMI), smoking habits, and alcohol intake. Most variables were re-categorised before analysis to increase statistical power and avoid problems from sparse data. Many of the factors were examined also in the earlier analysis of risk factors for high annual sickness absence [5].

Demographic variables, such as age at baseline and educational attainment were treated as fixed throughout the follow-up period. Others risk factors, such as work characteristics and health status, might change over the course of follow-up, and where information was available, were treated as time-varying. Further details are provided in Table 1, Table 2, Table 3, Table 4.

**Table 1:** Associations of early long-term economic inactivity with demographic variables. All demographic variables were derived from the baseline questionnaire and treated as fixed. Hazard ratios for each risk factor are adjusted only for age at baseline.

Risk factor	Men		Women	
	Number (%) with ELTEI	HR (95% CI)	Number (%) with ELTEI	HR (95% CI)
Age at baseline (years)				
50-54	65 (10.8%)	Ref	110 (14.3%)	Ref
55-59	182 (25.7%)	2.58 (1.94,3.43)	246 (27.6%)	2.07 (1.65,2.59)
60-65	57 (15.5%)	3.76 (2.58,5.47)	22 (19.5%)	3.70 (2.32,5.90)
Level of education				
No qualification/school only	81 (16.6%)	Ref	109 (18.8%)	Ref
Vocational training certificate	73 (13.0%)	0.77 (0.56,1.06)	112 (20.4%)	1.01 (0.78,1.32)
University degree or higher	150 (23.9%)	1.48 (1.13,1.95)	157 (24.4%)	1.32 (1.03,1.68)
General practice region				
London & South East	22 (21.0%)	Ref	42 (26.9%)	Ref
South Central & South West	99 (17.3%)	0.73 (0.46,1.16)	105 (18.8%)	0.73 (0.46,1.16)
East	63 (16.5%)	0.67 (0.41,1.09)	97 (22.6%)	0.67 (0.41,1.09)
West Midlands	44 (18.4%)	0.77 (0.46,1.28)	50 (22.5%)	0.77 (0.46,1.28)
North West & North East	76 (20.1%)	0.87 (0.54,1.39)	84 (20.6%)	0.87 (0.54,1.39)
Area deprivation score				
≤6 (most deprived)	135 (17.0%)	Ref	168 (20.7%)	Ref
7-8	99 (18.9%)	1.19 (0.92,1.54)	124 (22.6%)	1.11 (0.88,1.40)
9-10	70 (19.7%)	1.17 (0.88,1.57)	86 (20.8%)	1.04 (0.80,1.35)
Marital status				
Married/civil partnership/co-habiting	225 (17.5%)	Ref	279 (23.6%)	Ref
Single/widowed/divorced	79 (20.5%)	1.16 (0.90,1.50)	95 (16.4%)	0.65 (0.51,0.81)
Missing	–	–	4 (25.0%)	0.93 (0.35,2.50)

**Table 2:** Association of early long-term economic inactivity with aspects of personal finance and time demands outside paid work. Hazard ratios for each risk factor are adjusted only for age at baseline. At the time-points when a variable was not measured, the most recent available value was used.

Risk factor	Men		Women	
	Number (%) with ELTEI	HR (95% CI)	Number (%) with ELTEI	HR (95% CI)
Contribution to family income (baseline)				
Half or more	224 (16.7%)	Ref	190 (19.3%)	Ref
Between a quarter and a half	51 (23.0%)	1.41 (1.04,1.91)	97 (21.0%)	1.12 (0.88,1.43)
Less than a quarter	25 (33.3%)	2.48 (1.64,3.76)	83 (30.5%)	1.72 (1.33,2.23)
Missing	4 (10.3%)	0.66 (0.24,1.77)	8 (14.6%)	0.73 (0.36,1.49)
Housing tenure (baseline)				
Rented	20 (12.7%)	Ref	22 (12.6%)	Ref
Mortgaged	89 (12.7%)	0.95 (0.58,1.54)	134 (18.2%)	1.35 (0.86,2.11)
Owned outright or rent-free	193 (24.3%)	1.89 (1.19,2.99)	215 (26.0%)	1.92 (1.24,2.98)
Missing	2 (8.7%)	0.60 (0.14,2.55)	7 (18.4%)	1.32 (0.56,3.08)
Financial position (time-varying)				
Managing with difficulty	11 (5.1%)	Ref	21 (5.1%)	Ref
At least getting by	291 (5.7%)	1.06 (0.58,1.94)	357 (6.3%)	1.18 (0.76,1.83)
Missing	2 (6.1%)	1.19 (0.26,5.38)	–	–
Receive or expect private/employer pension (baseline)				
No	16 (9.0%)	Ref	66 (18.8%)	Ref
Expect to receive at/after SPA	32 (12.0%)	1.20 (0.65,2.21)	44 (16.1%)	0.79 (0.54,1.15)
Expect to receive before SPA	160 (21.8%)	2.55 (1.52,4.28)	186 (23.8%)	1.35 (1.02,1.79)
Already receive	95 (19.2%)	2.07 (1.21,3.54)	82 (23.6%)	1.22 (0.88,1.68)
Missing	1 (16.7%)	1.21 (0.16,9.11)	–	–
Expected total income after retirement compared to that at baseline (baseline)				
Less than a quarter	30 (8.1%)	Ref	103 (18.2%)	Ref
Between a quarter and a half	149 (20.0%)	2.56 (1.73,3.78)	157 (21.6%)	1.14 (0.89,1.47)
Half or more	110 (22.8%)	2.90 (1.94,4.35)	93 (27.3%)	1.46 (1.10,1.94)
Missing	15 (19.5%)	2.81 (1.51,5.23)	25 (17.6%)	1.07 (0.69,1.67)
Caring responsibilities (time-varying)				
None	254 (5.7%)	Ref	276 (5.9%)	Ref
Up to 20 hours/week	47 (5.9%)	1.04 (0.76,1.42)	89 (6.8%)	1.18 (0.93,1.50)
20 hours/week or more	3 (3.2%)	0.60 (0.19,1.88)	13 (6.2%)	0.92 (0.53,1.61)
Other unpaid work (time-varying)				
No	256 (5.4%)	Ref	309 (5.9%)	Ref
Yes	48 (7.0%)	1.36 (1.00,1.85)	69 (7.4%)	1.24 (0.95,1.61)

**Table 3:** Association of early long-term economic inactivity with variables relating to employment. Hazard ratios for each risk factor are adjusted only for age at baseline. All factors in this table are treated as time-varying.

Risk factor	Men		Women	
	Number (%) with ELTEI	HR (95% CI)	Number (%) with ELTEI	HR (95% CI)
Employment status				
Self-employed	52 (4.3%)	Ref	37 (4.6%)	Ref
Employed	252 (6.0%)	1.48 (1.10,1.99)	341 (6.4%)	1.43 (1.02,2.00)
Shift and/or night work				
Neither	247 (5.7%)	Ref	315 (6.2%)	Ref
Day shifts only	21 (4.0%)	0.71 (0.45,1.11)	40 (5.2%)	0.81 (0.58,1.13)
Night work	23 (5.4%)	0.96 (0.62,1.47)	14 (5.9%)	1.03 (0.60,1.76)
Missing	13 (10.2%)	1.67 (0.96,2.93)	9 (12.7%)	2.18 (1.12,4.23)
Physical demands of work				
Low	134 (6.7%)	Ref	198 (6.5%)	Ref
Intermediate	93 (5.6%)	0.86 (0.66,1.12)	135 (6.2%)	0.92 (0.74,1.14)
Heavy	72 (4.2%)	0.62 (0.46,0.82)	41 (4.4%)	0.67 (0.48,0.94)
Missing	5 (38.5%)	4.78 (1.94,11.80)	4 (28.6%)	3.71 (1.37,10.03)
Basis of payment				
Fixed salary	253 (5.9%)	Ref	330 (6.1%)	Ref
Paid by output	38 (3.8%)	0.60 (0.42,0.84)	38 (5.4%)	0.82 (0.58,1.14)
Missing	13 (11.6%)	1.72 (0.99,3.02)	10 (11.0%)	1.75 (0.93,3.28)
Annual holiday allowance				
More than 30 days	130 (6.6%)	Ref	155 (6.1%)	Ref
Up to 30 days	174 (5.1%)	0.70 (0.56,0.88)	223 (6.1%)	0.94 (0.77,1.16)
Difficult relationships at work and/or frequent job worries disturbing sleep				
None	146 (5.3%)	Ref	181 (5.6%)	Ref
Either	147 (5.8%)	1.16 (0.93,1.46)	187 (6.4%)	1.17 (0.95,1.43)
Missing	11 (12.4%)	2.41 (1.30,4.47)	10 (21.7%)	3.96 (2.08,7.52)
Rewarding work				
Yes	246 (5.5%)	Ref	315 (5.8%)	Ref
No	48 (5.9%)	1.09 (0.80,1.49)	54 (8.0%)	1.39 (1.04,1.86)
Missing	10 (13.0%)	2.31 (1.22,4.37)	9 (25.0%)	4.75 (2.44,9.25)
Friends at work				
Yes	95 (5.1%)	Ref	176 (5.4%)	Ref
No	199 (5.8%)	1.07 (0.84,1.36)	193 (6.7%)	1.19 (0.97,1.46)
Missing	10 (13.5%)	2.57 (1.34,4.95)	9 (23.7%)	4.76 (2.43,9.33)
Easily coping with both physical and mental demands of the job				
Yes	160 (5.3%)	Ref	152 (4.7%)	Ref
No	137 (5.8%)	1.10 (0.87,1.38)	220 (7.6%)	1.58 (1.29,1.95)
Missing	7 (18.0%)	3.12 (1.45,6.70)	6 (28.6%)	6.57 (2.89,14.93)
Overall job satisfaction				
Satisfied	262 (5.3%)	Ref	330 (5.8%)	Ref
Dissatisfied	32 (9.0%)	1.77 (1.23,2.56)	40 (9.6%)	1.59 (1.15,2.21)
Missing	10 (15.6%)	2.84 (1.51,5.36)	8 (23.5%)	4.22 (2.09,8.53)

**Table 4:** Association of early long-term economic inactivity with variables relating to health. Hazard ratios for each risk factor are adjusted only for age at baseline. Treated as time-varying unless differently specified.

Risk factor	Men		Women	
	Number (%) with ELTEI	HR (95% CI)	Number (%) with ELTEI	HR (95% CI)
Disabling pain in back, neck or limbs in past 12 months				
No	246 (5.6%)	Ref	274 (5.7%)	Ref
Yes	56 (5.8%)	1.04 (0.78,1.39)	103 (7.8%)	1.39 (1.11,1.74)
Missing	2 (7.1%)	1.27 (0.32,5.14)	1 (5.0%)	0.78 (0.11,5.60)
Depression				
No	237 (5.6%)	Ref	259 (5.8%)	Ref
Yes	64 (5.5%)	0.98 (0.75,1.30)	119 (7.0%)	1.19 (0.96,1.48)
Missing	3 (10.7%)	1.96 (0.63,6.12)	–	–
Somatising tendency (baseline)				
No	286 (18.0%)	Ref	324 (20.6%)	Ref
Yes	13 (18.8%)	1.21 (0.69,2.10)	43 (25.2%)	1.29 (0.94,1.77)
Missing	5 (23.8%)	1.26 (0.52,3.06)	11 (39.3%)	2.17 (1.19,3.97)
Sleep problems				
No severe symptoms	263 (5.5%)	Ref	297 (6.0%)	Ref
At least one severe symptom	41 (7.0%)	1.32 (0.95,1.83)	81 (6.9%)	1.18 (0.93,1.51)
Unintentional weight loss				
No	291 (5.7%)	Ref	357 (6.2%)	Ref
Yes	12 (4.2%)	0.74 (0.42,1.32)	21 (5.8%)	0.98 (0.63,1.52)
Missing	1 (5.3%)	0.73 (0.10,5.25)	–	–
Walking speed				
Easy pace/Fast	287 (5.4%)	Ref	348 (5.9%)	Ref
Very slow/Unable to walk	16 (15.2%)	2.42 (1.46,4.00)	29 (14.2%)	2.36 (1.61,3.45)
Missing	1 (7.1%)	1.27 (0.18,9.05)	1 (5.3%)	0.71 (0.10,5.05)
BMI (kg/m^2^) (baseline)				
<25	71 (15.0%)	Ref	167 (21.6%)	Ref
25-29.9	168 (21.5%)	1.45 (1.10,1.92)	100 (18.3%)	0.85 (0.66,1.09)
30-34.9	47 (16.0%)	1.11 (0.77,1.61)	59 (22.3%)	0.98 (0.73,1.32)
≥35	15 (18.1%)	1.15 (0.66,2.00)	38 (27.0%)	1.27 (0.89,1.81)
Missing	3 (7.1%)	0.48 (0.15,1.52)	14 (29.2%)	1.34 (0.78,2.32)
Alcohol intake (units per week) (baseline)				
≤1 unit	33 (17.4%)	Ref	92 (20.9%)	Ref
2-14 units	153 (18.0%)	0.95 (0.65,1.39)	225 (21.7%)	1.06 (0.83,1.35)
≥15	112 (19.9%)	1.06 (0.72,1.56)	27 (21.1%)	1.16 (0.75,1.78)
Missing	6 (8.5%)	0.46 (0.19,1.09)	34 (20.0%)	0.96 (0.65,1.42)
Smoking status (baseline)				
Never smoked	171 (18.5%)	Ref	232 (22.0%)	Ref
Ex-smoker	99 (17.4%)	0.95 (0.74,1.22)	121 (22.5%)	0.95 (0.76,1.18)
Current smoker	34 (19.4%)	1.06 (0.74,1.54)	24 (14.7%)	0.68 (0.44,1.03)
Missing	–	–	1 (5.9%)	0.21 (0.03,1.50)

### Statistical analysis

Analysis was based on participants who, at the time of recruitment, were in a paid job (including those on sick leave), would not reach their SPA for more than two years, and who subsequently completed at least the first two follow-up questionnaires.

Risk factors for ELTEI were explored by discrete-time Cox regression, taking as time intervals the periods between successive questionnaires. Follow-up of an individual was censored after the first time interval that met any of the following criteria: a) onset of ELTEI during the interval; b) interval ended less than two years before SPA (because by definition they would not be at risk for onset of ELTEI throughout the next interval); or c) no questionnaire completed two years after end of interval (because this would make it impossible to be sure that a period of economic inactivity which began in the next interval extended beyond 12 months). Thus, a person could be at risk for a maximum of five follow-up intervals, the last ending at the fifth follow-up. Associations were summarised by hazard ratios (HRs) with 95% confidence intervals (CIs). For each time interval, time-varying risk factors were classified according to their most recent known value at the start of the interval. All analyses were conducted separately for men and women. This strategy was specified a priori because work circumstances and pathways out of employment are known to differ by sex [12,13].

Risk estimates were derived initially with adjustment only for age at baseline. Significant risk factors in the minimally adjusted models (95% confidence interval excluding 1) were then included in a mutually adjusted model. Where there was a possibility that an important risk factor was omitted from the final model because its effect was obscured by negative confounding in the analysis adjusted only for age, that was checked by adding it to the mutually adjusted model. Finally, as a sensitivity exercise, the mutually adjusted model was re-run after exclusion of participants with imputed dates of starting or leaving jobs. All analyses were conducted with Stata statistical software (version 17.0) [14]. Potential collinearity among covariates was assessed using variance inflation factors (VIFs) calculated from a linear regression model containing the same predictors. No evidence of high multicollinearity was apparent. Tests of the proportional hazards assumption in the final models were based on Schoenfeld residuals.

Written consent was obtained from participants. This study complies with the Declaration of Helsinki. Ethical approval was obtained from the National Health Service (NHS) Research Ethics Committee North West-Liverpool East, UK (REC reference 12/NW/0500).

## Results

Of the 8,134 participants recruited to the HEAF study, 2,427 were excluded because they had already reached their SPA before completing the baseline questionnaire (N = 1,622) or would do so in less than two years (N = 805). Among the remaining 5,707, 1,148 were excluded as they were not in a paid job at baseline, 1,058 because they did not complete both of the first two follow-up questionnaires, and a further 51 with missing information on dates of starting or finishing jobs that could not satisfactorily be imputed. This left 3,450 participants (1,676 men and 1,774 women) who met our criteria for analysis, including 469 for whom at least one job date was imputed.

The 3,450 participants contributed usable information on a total of 11,561 follow-up intervals. They included 682 individuals (19.8%) who experienced onset of ELTEI while under follow-up. Of those, 208 (30.5%) reported that the job loss leading to ELTEI was at least partly related to health, while the remaining 474 (60.5%) said that it was not. Among the 2,768 with no ELTEI, 539 completed all follow-ups, while 2,229 dropped out before the last time interval because they failed to return a questionnaire (N = 1,713) or came within two years of SPA (N = 516).

Among participants who were at risk of ELTEI when they completed the baseline questionnaire, those with maximal follow-up data (i.e., follow-up not censored because of failure to return a questionnaire) tended to have higher educational attainment than those with no usable follow-up information, to report better self-rated health, and less often to be a current smoker. They were also less likely to reside in more deprived areas (S2 Table). However, the baseline characteristics of participants with maximal follow-up and those whose follow-up was censored because of failure to return a questionnaire were generally similar. Times between onset of ELTEI and SPA ranged from 1 to 9.1 years, with median 3.2 years and mean 3.5 years. ELTEI occurred more frequently among women than men (378 (21%) v 304 (18%)); and among participants aged 55–59 years at recruitment as compared with those aged 50–54 or 60–65 (ELTEI at baseline age 50–54: 13%; 55–59: 27%; 60–65: 16%), but it should be noted that time at risk differed importantly by age at baseline.

Table 1 shows associations of ELTEI with demographic variables. When time at risk was considered, ELTEI was more likely in men and women who were older at baseline, and also in those with higher levels of education. No significant associations were found with area deprivation score or general practice region. However, women who were single, divorced or widowed were less likely to experience ELTEI than those who were married or co-habiting.

Table 2 summarises associations of ELTEI with various aspects of personal finance. Both in men and women, the outcome occurred more frequently in those who contributed less than a quarter to the total family income, and in those whose property was owned outright or rent-free. Among men, those who either expected or already received a private or employer’s pension before SPA, were more than twice as likely to experience ELTEI as those who did not, and ELTEI was also more likely in those who expected a smaller reduction in their income after retirement. In women, these associations were present but weaker. Caring responsibilities were not related to ELTEI in either sex.

For fixed covariates, the prevalence of ELTEI is based on number of participants (N = 3,450) whereas for time varying covariates, the prevalence is based on follow-up intervals (N = 11,561)

The strongest work-related predictors of ELTEI were being employed rather than self-employed, having work that was less demanding physically, and job dissatisfaction (Table 3). Among women, not feeling rewarded at work and struggling to cope with job demands were additional risk factors. In men, having a lower annual holiday allowance and being paid by output both carried a lower risk of ELTEI.

The prevalence of ELTEI is based on follow-up intervals (N = 11,561)

As shown in Table 4, the only measures of health clearly associated with subsequent ELTEI were report of very slow walking speed and (in women only) disabling musculoskeletal pain.

For fixed covariates, the prevalence of ELTEI is based on number of participants (N = 3,450) whereas for time varying covariates, the prevalence is based on follow-up intervals (N = 11,561)

Table 5 presents mutually adjusted risk estimates from regression models that included selected variables showing associations in the models that adjusted only for age at baseline. In men, the strongest predictors of ELTEI were older age at baseline, lower contribution to family income, having no mortgage or rent commitment, expecting or already receiving a private or employer’s pension before SPA, expecting a smaller reduction in income following retirement, job dissatisfaction and report of very slow walking speed. In women, associations were similar, although generally somewhat weaker than in men. In addition, risk was significantly elevated in women who were married or co-habiting, and in those who reported that they were not coping easily with the physical and mental demands of their job.

**Table 5:** Mutually adjusted associations of early long-term economic inactivity with selected variables.

	Men	Women
	HR (95% CI)	HR (95% CI)
Age at baseline		
50-54	Ref	Ref
55-59	2.57 (1.92,3.43)	2.07 (1.64,2.61)
60-65	4.61 (3.01,7.07)	3.93 (2.40,6.44)
Level of education		
No qualification/school only	Ref	Ref
Vocational training certificate	0.85 (0.62,1.17)	1.01 (0.77,1.32)
University degree or higher	1.30 (0.96,1.75)	1.25 (0.97,1.62)
Marital status		
Married/civil partnership/co-habiting	Ref	Ref
Single/widowed/divorced	1.30 (1.00,1.70)	0.68 (0.53,0.88)
Missing		0.83 (0.31,2.26)
Contribution to family income (baseline)		
Half or more	Ref	Ref
Between a quarter and a half	1.43 (1.05,1.95)	1.01 (0.78,1.31)
Less than a quarter	2.06 (1.30,3.25)	1.66 (1.25,2.19)
Missing	1.25 (0.30,5.19)	0.55 (0.17,1.83)
Housing tenure (baseline)		
Rented	Ref	Ref
Mortgaged	0.94 (0.57,1.55)	1.21 (0.76,1.94)
Owned outright or rent-free	1.75 (1.09,2.83)	1.62 (1.02,2.56)
Missing	0.38 (0.05,2.95)	2.46 (0.64,9.41)
Receive or expect private/employer pension (baseline)		
No	Ref	Ref
Expect to receive at/after SPA	1.02 (0.55,1.91)	0.68 (0.46,1.01)
Expect to receive before SPA	2.18 (1.27,3.77)	1.17 (0.87,1.59)
Already receive	1.72 (0.98,3.02)	1.09 (0.78,1.54)
Missing	1.84 (0.23,14.64)	–
Expected total income after retirement compared to that at baseline (baseline)		
Less than half	Ref	Ref
Between a quarter and a half	2.20 (1.48,3.29)	1.16 (0.90,1.49)
Half or more	2.27 (1.50,3.44)	1.39 (1.04,1.85)
Missing	2.88 (1.51,5.51)	1.40 (0.90,2.20)
Other unpaid work (time-varying)		
No	Ref	Ref
Yes	1.16 (0.85,1.60)	1.21 (0.92,1.58)
Employment status		
Self-employed	Ref	Ref
Employed	1.54 (1.08,2.10)	1.63 (1.14,2.33)
Physical demands of work		
Low	Ref	Ref
Intermediate	0.97 (0.74,1.28)	0.94 (0.75,1.18)
Heavy	0.82 (0.60,1.13)	0.68 (0.48,0.97)
Missing	5.02 (1.71,14.76)	1.23 (0.34,4.57)
Easily coping with both physical and mental demands of the job		
Yes	–	Ref
No	–	1.52 (1.21,1.92)
Missing	–	2.79 (0.66,11.77)
Overall job satisfaction		
Satisfied	Ref	Ref
Dissatisfied	1.81 (1.24,2.64)	1.40 (0.99,1.99)
Missing	1.35 (0.60,4.67)	3.15 (0.98,10.10)
Disabling pain in back, neck or limbs in past 12 months		
No		Ref
Yes		1.25 (0.98,1.61)
Missing		0.88 (0.12,6.36)
Depression		
No		Ref
Yes		1.07 (0.84,1.36)
Missing		–
Walking speed		
Normal/Fast	Ref	Ref
Very slow/Unable to walk	3.17 (1.89,5.33)	1.77 (1.18,2.69)
Missing	1.19 (0.16,8.52)	0.76 (0.10,5.64)

There was no evidence of violation of the proportional hazards assumption in any of the models.

To explore the possibility that an important effect of depression or musculoskeletal pain might have been missed in men because of inverse associations with personal financial security, we repeated the analysis for Table 5 including depression and regional pain in the model for men. However, that did not indicate any negative confounding of note.

In a sensitivity analysis that excluded participants with imputed dates, risk estimates were generally similar or slightly higher (S3 Table).

## Discussion

Among the men whom we studied, the main determinants of ELTEI appear to have been financial. It was most frequent in those who could more easily afford to give up paid work, as indicated by a smaller contribution to family income, eligibility for a private or employer’s pension before SPA, expecting a smaller reduction in income after retirement and no mortgage or rent commitment. Job dissatisfaction appeared also to be a factor, but the only measure of health that predicted ELTEI was report of a very slow walking speed. In women, financial security again seems to have been the main influence on ELTEI, although less strongly than in men.

We conducted our analysis as part of a broader investigation into determinants of reduced work participation (and therefore productivity) in late middle age. We reasoned that the two major contributions to reduced participation in work would be from high annual sickness absence while people were in paid employment, and from prolonged periods without a paid job. We explored risk factors for the first of these outcomes in an earlier paper [5], and here focus on the second. We based our outcome on a continuous period of 12 months or longer without paid work because it indicated a major loss of individual productivity and was ascertainable from the HEAF study with its baseline questionnaire and subsequent annual follow-ups. Furthermore, it is consistent with measures used in other studies [15], and with UK Government definition of long-term worklessness, which includes individuals either unemployed or economically inactive for at least 12 months [16]. In comparison, self-reported retirement would have been less satisfactory because it depends on the expectations of the participant when he/she provides information. An individual reporting himself to be retired might unexpectedly return to paid work within a couple of months, making the impact on productivity relatively small. On the other hand, another participant might be out of work for longer than a year, with a bigger effect on productivity, but not be counted as retired because at the time of answering a questionnaire, he was still trying to obtain further employment.

Major strengths of the HEAF study are its longitudinal design and its ascertainment of a wide range of factors relating to employment, health, personal finance, and time commitments outside work which might plausibly impact on early exit from paid work. The HEAF cohort was recruited from the lists of a geographically dispersed set of general practices with eligibility for inclusion based solely on age. The incomplete participation of those invited to join the study (21%) leaves open the possibility that the final study sample was in some respects importantly unrepresentative of the national population. For example, there may have been over-representation of people with higher levels of education and employed in non-manual jobs. Furthermore, participants who contributed to the analysis of risk factors for ELTEI differed from those who were at risk of ELTEI at baseline but failed to provide any usable follow-up data. They tended to have a higher educational attainment, report better self-rated health and less often be current smokers. However, we have no reason to expect that in participants who failed to provide usable follow-up, associations of ELTEI with risk factors would have been substantially different from those that we found using the data that were available.

We acknowledge our use of missing-value categories as a potential limitation. We chose this approach because the proportion of missing data across variables was small, and it allowed us to retain a maximum sample size without relying on the assumptions required for multiple imputation. It is possible that the missing-indicator approach introduced bias if missingness itself was informative, but given the low prevalence of missing data, any such bias is likely to have been small.

The prospective design meant that ascertainment of risk factors preceded the outcome, and as such cannot have been influenced by it. This would tend to render any errors in reporting of risk factors non-differential with respect to the outcome, biasing risk estimates towards the null. Similarly, any changes in a time-varying risk factor between its assessment at the start of a follow-up interval and an onset of ELTEI later in the same interval might be expected to attenuate risk estimates. The sensitivity analysis in S3 Table indicates that missing information about dates of job changes did not spuriously inflate risk estimates.

Within the period of study, 19.8% of participants experienced ELTEI. This will be an underestimate of lifetime prevalence because some (particularly among those who were younger at entry to the study) were still at risk at the end of follow-up. Statistics from the Office for National Statistics indicate a 26% prevalence of economic inactivity among people aged 50–64 years [17], although not all of that will have been long-term.

The strong association of ELTEI with age at entry to the study tallies with the observation that most ELTEI occurred less than four years before SPA (median interval 3.2 years). SPA varied by sex and date of birth, but at the end of follow-up, everyone aged 50–54 at baseline would have come this close to their SPA. The lower incidence of ELTEI at longer intervals before SPA is likely in large part to reflect the greater financial challenge of leaving paid work so early. Private and employers’ pensions, if available, are often reduced actuarially when drawn at younger ages.

The association of early retirement with financial resilience accords with an earlier report that, for example, owning a home without a mortgage is a strong predictor of economic inactivity [18]. Such resilience might be expected to occur most often among higher earners in white collar occupations. It appears that recently workers with intermediate levels of education, and in lower paying industries and occupations have experienced the largest increases in economic inactivity, but that included inactivity of shorter duration, and followed the emergence Covid-19 [19].

It is perhaps not surprising that report of job dissatisfaction predicted ELTEI, and one might expect its impact to be greatest among those who can most easily afford to retire early. It carried a higher HR than more specific measures such as unrewarding work and an absence of friends at work. In contrast to our findings on high annual sickness absence [5], heavy physical work was not a predictor, if anything being associated with a reduced risk (Table 3). This inverse relationship may have occurred in part because manual workers less often had the financial security to retire early. Caring and other non-occupational time commitments seem not to have been a major factor in early retirement.

Whereas high sickness absence was strongly predicted by musculoskeletal illness, depression and determinants of chronic disease such as obesity and smoking [5], the only measure of health predictive of ELTEI was report of a very slow walking speed (a fairly rare symptom). Previous analysis of the HEAF study has demonstrated that, independently of confounders, both men and women with a self-reported very slow walking speed were four times as likely to experience job loss for health reasons as those reporting normal walking speed [20]. The absence of association between ELTEI and unintentional weight loss may be because the latter is often a marker for severe acute illness which either responds to treatment or is fairly rapidly fatal. It appears that depression and painful musculoskeletal disorders, while important causes of prolonged sickness absence, did not frequently lead to ELTEI.

Within the HEAF study, we have previously explored determinants of health-related job loss, defined as job loss of any duration which the participant attributed at least partly to ill-health [21,22]. Those analyses identified aspects of health such as musculoskeletal pain and common mental health conditions, as significant predictors of leaving work for perceived health reasons. The present analysis examined a conceptually distinct outcome and suggests that such findings cannot necessarily be extrapolated to onset of economic inactivity that is longer term and not necessarily perceived by the individual as health-related.

The picture that emerges from this and our related report on high annual sickness absence is that while sickness absence and ELTEI are both important causes of reduced productivity at older ages, their determinants are rather different. Economic inactivity seems to be driven predominantly by considerations of personal finance and affordability and is more common in people with higher levels of education and in occupations that are less demanding physically. In contrast, high sickness absence occurs most frequently in those from poorer social circumstances with less education, poor personal health, and jobs that are more demanding physically. In other words, the people whom increases in SPA most often oblige to work to older ages tend to be those who will find it most difficult to do so. Because of their lower average level of education, they will often also have started productive employment at a younger age.

### Strengths and limitations of this study

1. People invited to take part in the study should have been broadly representative of the national population.
2. Major strengths of the study were its longitudinal design and its ascertainment of a wide range of factors which might plausibly impact on early exit from paid work.
3. Although we did not assess whether participants with ELTEI ever sought further work, the outcome directly reflected impact on economic output.
4. Because it was derived from a limited duration of follow-up, the prevalence of ELTEI that we recorded is likely to underestimate lifetime prevalence.

## Supporting information

S1 Table HEAF questions, response categories, coded analysis variables and reference categories. (DOCX)

S2 Table Frequency and (in brackets) prevalence (%) of baseline characteristics among participants who were at risk of ELTEI when they completed the baseline questionnaire according to completeness of subsequent follow-up. (DOCX)

S3 Table Mutually adjusted associations of early long-term economic inactivity with selected variables in participants with no imputed dates (N = 2,981). (DOCX)
