# Supplementary material for: Associations of sociodemographic, health, and work-related factors with early long-term economic inactivity in older workers: Findings from the Health and Employment After Fifty study
Source: PLoS One. 2026 Oct 6;21(10):e0359865. doi: 10.1371/journal.pone.0359865 (PMC13641384; doi:10.1371/journal.pone.0359865)
Supplement: Supplementary file 1 [file pone.0359865.s001.docx]

S1 Table. HEAF questions, response categories, coded analysis variables and reference categories

| **Question** | **Response categories** | **Classification for analysis** |
| --- | --- | --- |
| Do you have any of the following qualifications? | o/gcse levels; a levels; vocational cert; university degree; higher professional qualification | no qualifications/school level vs vocational training certificate vs university degree/higher |
| General practice region | London; South East; South Central; South West; East; West Midlands; North West; North East | London & South East vs South Central & South West vs East vs West Midlands vs North West & North East |
| What is your current marital status? | married; single; civil partnership; widowed; divorced | married/civil partnership vs single/widowed/divorced |
| Area-level deprivation | English Index of Multiple Deprivation 2010 [1] | ≤ 6 (most deprived) vs 7-8 vs  9-10 (least deprived) |
| How much of the household income comes from money you earn in a paid job? | none; less than a quarter; between a quarter and a half; half or more | half or more vs between a quarter and half vs less than a quarter |
| Is your home? | owned outright by you; owned by you with mortgage; rented; rent free | owned outright by you/rent free vs owned by you with mortgage vs rented |
| How well do you feel you are managing financially these days? | living comfortably/doing alright; just about getting by; finding it difficult/very difficult to make ends meet | at least getting by vs managing with difficulties |
| Apart from any state pension do you currently receive a private or employer's pension? | no; yes | combined to: no access vs expect to receive such a pension at/after SPA vs expect to receive one before SPA vs already receive one |
| Do you expect to receive a private or employer's pension in the future? | no; yes |  |
| When you are fully retired and receiving any pensions that are due to you, how will your total personal income compare with what you get now? | less than a quarter; between a quarter and half; half or more |  |
| In an average week, outside work, roughly how many hours would you spend doing the following activities? | giving personal care to someone in your home or family | none vs up to 20 hours/week vs 20 hours a week or more |
|  | working in an unpaid job for others outside your home and family (e.g. as a volunteer for a charity) | yes vs no |
| *Work-related variables* | | |
| Which of the following best describes your present work situation? | employed; self-employed; unemployed; retired | employed; self-employed (unemployed and retired excluded from the current analysis) |
| Does your job involve rotating or variable shifts? | often; sometimes; rarely/never | combined to: neither vs day shifts only vs night work |
| Does your main job involve night work? | often; sometimes; rarely/never |  |
| In your main job, does an average day at work involve any of the following activities? (all response options No/Yes) | does the job involve kneeling or squatting for longer than 1 hour per day | low where the participant reported that their job did not entail hard physical work or involve any of the specified list of activities that could be physically demanding; intermediate where they reported exposure to at least one such activity, but that their job did not involve hard physical work; and heavy where they said that their job involved hard physical work. |
|  | does the job involve climbing a ladder |  |
|  | does the job involve digging or shovelling |  |
|  | does the job involve lifting weights of 10kg or more by hand |  |
|  | does the job involve standing or walking for more than 3 hours at a time |  |
|  | does the job involve hard physical work that makes you hot or sweaty |  |
| Ignoring overtime, does your main job give you a fixed salary, or are you paid according to your output (e.g. the number of tasks you do or things you make)? | fixed salary; paid by output |  |
| How much holiday are you allowed from your job per year (including Bank Holidays)? | open question | up to 30 days vs more than 30 days |
| Is there anyone at work you find very difficult to get on with? | yes; no | combined as “Difficult relationship at work” if either they are often criticised unfairly at work or there is someone at work they find very difficult to get on with |
| Do you ever get criticised unfairly at work? | often; sometimes; rarely/never |  |
| Do you ever lie awake at night worrying about work or angry about work? | often; sometimes; rarely/never | combined with the previous one to derive “Difficult relationships at work and/or job worries”. Present if either difficult relationships at work or often lie awake at night worrying about work |
| Does your work give you a feeling of achievement? | often; sometimes; rarely/never | rewarding work defined as: often/sometimes appreciated AND often/sometimes feeling of achievement |
| In your work, do you feel appreciated by others? | often; sometimes; rarely/never |  |
| Do you have friends at work with whom you also spend time outside work? | Yes; no |  |
| Currently, how well do you cope with the physical demands of your job | easily; with some difficulty; with great difficulty; not coping | easily coping with both mental and physical demands of the job vs at least some difficulty with mental or physical demands of job |
| Currently, how well do you cope with the mental demands of your job | easily; with some difficulty; with great difficulty; not coping |  |
| How satisfied are you with your job as a whole? | very satisfied; satisfied; dissatisfied; very dissatisfied | very satisfied/satisfied vs dissatisfied/very dissatisfied |
| *Health behaviour and health-related variables* | | |
| Please give your height and your weight | height reported in feet and inches, or centimetres weight reported in stones and pounds, or kilograms | converted to height in metres and weight in kilograms. Body mass index (BMI) in kg/m^2^ was calculated as self-reported weight in kilograms, divided by the square of self-reported height in metres, and categorised as: normal/underweight (<25) vs overweight (25-29.9) vs obese (30-34.9) vs severely obese (≥35) |
| How much of the following do you drink per week, on average? | beer, cider, lager (pints); wine, sherry (glasses); spirits, liqueurs (measures) | converted to units of alcohol per week and categorised: low/non drinker (≤1); moderate (2-14); heavy (15+) |
| Have you ever smoked regularly? If yes, do you still smoke regularly? | no; yes response categories for both questions | smoking status coded as: never; ex; current |
| How much have you been troubled by sleep problems in the past 3 months? (all as: no problem; mild problem; moderate problem; severe problem) | difficulty falling asleep | sleep problems (severe symptoms on ≥1 sleep item) [2] |
|  | difficulty staying asleep |  |
|  | waking up too early |  |
|  | not feeling refreshed in the morning |  |
| Thinking about your weight, in the past 12 months have you lost more than 10 pounds (4.5 kg) unintentionally (i.e. without dieting or exercise)? | yes; no |  |
| Which of the following best describes your walking speed? | unable to walk; very slow; stroll at an easy pace; normal pace; fairly brisk; fast | stroll at an easy pace to fast vs unable to walk/very slow |
| During the past 12 months, have you had pain in your BACK or NECK for a month or longer that made it difficult or impossible to get washed or dressed or do household chores? | yes; no | disabling musculoskeletal pain at ≥1 anatomical site  vs no disabling musculoskeletal pain at any of the sites |
| During the past 12 months, have you had pain in your ARM(S) or SHOULDER(S) for a month or longer that made it difficult or impossible to get washed or dressed or do household chores? | yes; no |  |
| During the past 12 months, have you had pain in your LEG(S) for a month or longer that made it difficult or impossible to get washed or dressed or do household chores? | yes; no |  |
| Depressive symptoms assessed with the CES-D scale [3] |  | ≥ 16 (depressed) vs <16 (not depressed) |
| Below is a list of problems that people sometimes have. Please read each one carefully and tick the box that best describes how much that problem has distressed or bothered you during the past 7 days including today (all as: not at all; a little bit; moderately; quite a bit; extremely) | faintness or dizziness | somatising tendency (≥2 symptoms rated at least moderately distressing) [4] |
|  | pains in the heart or chest |  |
|  | nausea or upset stomach |  |
|  | trouble getting your breath |  |
|  | hot or cold spells |  |

1.	Department for Communities and Local Government. The English Indices of Deprivation 2010. London; 2011.

2.	Jenkins CD, Stanton BA, Niemcryk SJ, Rose RM. A scale for the estimation of sleep problems in clinical research. J Clin Epidemiol. 1988;41(4):313–21.

3.	Radloff LS. The CES-D Scale:A Self-Report Depression Scale for Research in the General Population. Applied Psychological Measurement. 1977;1(3):385–401.

4.	Derogatis LR, Melisaratos N. The Brief Symptom Inventory: an introductory report. Psychol Med. 1983;13(3):595–605.
