# Supplementary material for: Associations of sociodemographic, health, and work-related factors with early long-term economic inactivity in older workers: Findings from the Health and Employment After Fifty study
Source: PLoS One. 2026 Oct 6;21(10):e0359865. doi: 10.1371/journal.pone.0359865 (PMC13641384; doi:10.1371/journal.pone.0359865)
Supplement: Supplementary file 2 [file pone.0359865.s002.docx]

S2 Table. Frequency and (in brackets) prevalence (%) of baseline characteristics among participants who were at risk of ELTEI when they completed the baseline questionnaire according to completeness of subsequent follow-up.

|  | **Follow-up intervals with usable information** | | |
| --- | --- | --- | --- |
|  | **None**  **(N=1,109)** | **≥1 but censored because failed to return a questionnaire**  **(N=1,713)** | **Maximal follow-up**  **(N=1,737)** |
| Sex |  |  |  |
| Male | 632 (57.0) | 775 (45.2) | 901 (51.9) |
| Female | 477 (43.0) | 938 (54.8) | 836 (48.1) |
| ^a^Age at baseline (years) |  |  |  |
| 50-54 | 489 (44.1) | 1369 (39.7) | |
| 55-59 | 472 (42.6) | 1600 (46.4) | |
| 60-65 | 148 (13.4) | 481 (13.9) | |
| Level of education |  |  |  |
| No qualification/school only | 412 (37.2) | 523 (30.5) | 544 (31.3) |
| Vocational training certificate | 376 (33.9) | 535 (31.2) | 577 (33.2) |
| University degree or higher | 321 (28.9) | 655 (38.2) | 616 (35.5) |
| Marital status |  |  |  |
| Married/civil partnership/co-habiting | 742 (66.9) | 1229 (71.8) | 1240 (71.4) |
| Single/widowed/divorced | 358 (32.3) | 475 (27.7) | 489 (28.2) |
| Missing | 9 (0.8) | 9 (0.5) | 8 (0.5) |
| Area deprivation score |  |  |  |
| ≤6 (most deprived) | 592 (53.4) | 789 (46.1) | 819 (47.2) |
| 7-8 | 309 (27.9) | 526 (30.7) | 548 (31.6) |
| 9-10 | 208 (18.8) | 398 (23.2) | 370 (21.3) |
| Self-rated health |  |  |  |
| At least good | 833 (75.1) | 1436 (83.8) | 1425 (82.0) |
| Fair/poor | 254 (22.9) | 265 (14.5) | 294 (16.9) |
| Missing | 22 (2.0) | 12 (0.7) | 18 (1.0) |
| Smoking status |  |  |  |
| Never smoked | 533 (48.1) | 1010 (59.0) | 969 (55.8) |
| Ex-smoker | 378 (34.1) | 508 (30.0) | 598 (34.4) |
| Current smoker | 186 (16.8) | 181 (10.6) | 157 (9.0) |
| Missing | 12 (1.1) | 14 (0.8) | 13 (0.8) |
| BMI (kg/m^2^) |  |  |  |
| <25 | 325 (29.3) | 632 (36.9) | 618 (35.6) |
| 25-29.9 | 451 (40.7) | 666 (38.9) | 663 (38.2) |
| 30-34.9 | 192 (17.3) | 268 (15.7) | 289 (16.6) |
| ≥35 | 110 (9.9) | 104 (6.1) | 120 (6.9) |
| Missing | 31 (2.8) | 43 (2.5) | 47 (2.7) |

^a^ When analysing age distributions, we combined all participants with any follow-up because the potential for censoring for failure to complete a questionnaire would be lower in those close to their SPA at baseline.
