# Supplementary material for: Associations of sociodemographic, health, and work-related factors with early long-term economic inactivity in older workers: Findings from the Health and Employment After Fifty study
Source: PLoS One. 2026 Oct 6;21(10):e0359865. doi: 10.1371/journal.pone.0359865 (PMC13641384; doi:10.1371/journal.pone.0359865)
Supplement: Supplementary file 3 [file pone.0359865.s003.docx]

**S3 Table. Mutually adjusted associations of early long-term economic inactivity with selected variables in participants with no imputed dates (N=2,981)**

|  | **Men** | **Women** |
| --- | --- | --- |
|  | **HR (95% CI)** | **HR (95% CI)** |
| Age at baseline |  |  |
| 50-54 | Ref | Ref |
| 55-59 | 2.99 (2.13,4.19) | 2.35 (1.81,3.06) |
| 60-65 | 6.08 (3.70,10.00) | 5.38 (3.18,9.07) |
| Level of education |  |  |
| No qualification/school only | Ref | Ref |
| Vocational training certificate | 0.86 (0.60,1.23) | 0.99 (0.74,1.33) |
| University degree or higher | 1.26 (0.90,1.76) | 1.42 (1.07,1.89) |
| Marital status |  |  |
| Married/civil partnership/co-habiting | Ref | Ref |
| Single/widowed/divorced | 1.34 (0.99,1.83) | 0.60 (0.45,0.80) |
| Missing | - | 0.97 (0.35,2.64) |
| Contribution to family income (baseline) |  |  |
| Half or more | Ref | Ref |
| Between a quarter and a half | 1.71 (1.21,2.42) | 0.94 (0.71,1.26) |
| Less than a quarter | 1.59 (0.80,3.18) | 1.59 (1.16,2.17) |
| Missing | 1.39 (0.18,10.62) | 0.74 (0.19,2.81) |
| Housing tenure (baseline) |  |  |
| Rented | Ref | Ref |
| Mortgaged | 1.09 (0.58,2.06) | 1.66 (0.90,3.06) |
| Owned outright or rent-free | 2.30 (1.26,4.23) | 2.50 (1.37,4.56) |
| Missing | 0.58 (0.05,7.16) | 2.50 (0.52,11.91) |
| Receive or expect private/employer pension (baseline) |  |  |
| No | Ref | Ref |
| Expect to receive at/after SPA | 1.01 (0.42,2.40) | 0.72 (0.47,1.12) |
| Expect to receive before SPA | 2.36 (1.06,5.25) | 1.38 (0.97,1.96) |
| Already receive | 1.68 (0.75,3.77) | 1.21 (0.82,1.79) |
| Missing | 2.31 (0.28,19.35) | - |
| Expected total income after retirement compared to that at baseline (baseline) |  |  |
| Less than half | Ref | Ref |
| Between a quarter and a half | 2.36 (1.50,3.72) | 1.15 (0.88,1.52) |
| Half or more | 2.22 (1.38,3.57) | 1.41 (1.03,1.93) |
| Missing | 1.16 (0.39,3.51) | 1.29 (0.75,2.20) |
| Other unpaid work (time-varying) |  |  |
| No | Ref | Ref |
| Yes | 1.18 (0.79,1.75) | 1.20 (0.88,1.62) |
| Employment status |  |  |
| Self-employed | Ref | Ref |
| Employed | 2.50 (1.55,4.03) | 2.12 (1.33,3.39) |
| Physical demands of work |  |  |
| Low | Ref | Ref |
| Intermediate | 1.04 (0.76,1.41) | 1.00 (0.78,1.28) |
| Heavy | 0.78 (0.54,1.14) | 0.68 (0.46,1.01) |
| Missing | - | - |
| Easily coping with both physical and mental demands of the job |  |  |
| Yes | - | Ref |
| No | - | 1.81 (1.41,2.34) |
| Missing | - | 2.81 (0.30,26.65) |
| Overall job satisfaction |  |  |
| Satisfied | Ref | Ref |
| Dissatisfied | 2.03 (1.34,3.07) | 1.39 (0.95,2.04) |
| Missing | 1.39 (0.32,6.04) | 2.02 (0.41,9.98) |
| Disabling pain in back, neck or limbs in past 12 months |  |  |
| No | - | Ref |
| Yes | - | 1.21 (0.92,1.60) |
| Missing | - | 0.98 (0.14,7.11) |
| Depression |  |  |
| No | - | Ref |
| Yes | - | 1.01 (0.77,1.32) |
| Missing | - | - |
| Walking speed |  |  |
| Normal/Fast | Ref | Ref |
| Very slow/Unable to walk | 2.89 (1.58,5.28) | 1.94 (1.25,3.02) |
| Missing | 1.32 (0.18,9.55) | 0.92 (0.12,6.88) |
