# Supplementary material for: Associations of sociodemographic, health, and work-related factors with early long-term economic inactivity in older workers: Findings from the Health and Employment After Fifty study
Source: PLoS One. 2026 Oct 6;21(10):e0359865. doi: 10.1371/journal.pone.0359865 (PMC13641384; doi:10.1371/journal.pone.0359865)
Supplement: Supplementary file 4 [file pone.0359865.s005.docx]

August 2026

Dear Editors

**Re: S. D’Angelo, G. Ntani, I. Madan, D. Coggon.**

***Associations of sociodemographic, health, and work-related risk factors with early long-term economic inactivity in older workers: findings from the Health and Employment After Fifty study***

Many thanks for your reviewers’ comments regarding our manuscript. I am pleased to report that we have been able to address these in a modified manuscript (in track changes mode). We have detailed the changes made below.

With all best wishes

**Dr Stefania D’Angelo**

**Senior Research Fellow and Epidemiologist**

This an interesting study with appropriate design but needs a strong and rubout  analysis , it is necessary to  revise some aspect of analysis as revivers  have mentioned in their comments .

Journal Requirements:
When submitting your revision, we need you to address these additional requirements.
1. Please ensure that your manuscript meets PLOS ONE's style requirements, including those for file naming. The PLOS ONE style templates can be found at
[https://journals.plos.org/plosone/s/file?id=wjVg/PLOSOne_formatting_sample_main_body.pdf](https://eur03.safelinks.protection.outlook.com/?url=https%3A%2F%2Ftrack.editorialmanager.com%2FCL0%2Fhttps%3A%252F%252Fjournals.plos.org%252Fplosone%252Fs%252Ffile%253Fid%3DwjVg%252FPLOSOne_formatting_sample_main_body.pdf%2F1%2F010f019f1bc4a654-a34a84a7-1535-49a9-8388-3d7f80682798-000000%2FeoRPp4dvArCRHZ14MuaCeNk2YHFQDVngrnNJ4dLevpo%3D258&data=05%7C02%7Csd%40mrc.soton.ac.uk%7Ca6e94d276ea9414ace5d08ded722aa5d%7C4a5378f929f44d3ebe89669d03ada9d8%7C0%7C0%7C639184741380426013%7CUnknown%7CTWFpbGZsb3d8eyJFbXB0eU1hcGkiOnRydWUsIlYiOiIwLjAuMDAwMCIsIlAiOiJXaW4zMiIsIkFOIjoiTWFpbCIsIldUIjoyfQ%3D%3D%7C0%7C%7C%7C&sdata=tIz5h6OFpCfC6C3C6piOWLRFlWJu716aPLA%2FZ3xd63s%3D&reserved=0) and [https://journals.plos.org/plosone/s/file?id=ba62/PLOSOne_formatting_sample_title_authors_affiliations.pdf](https://eur03.safelinks.protection.outlook.com/?url=https%3A%2F%2Ftrack.editorialmanager.com%2FCL0%2Fhttps%3A%252F%252Fjournals.plos.org%252Fplosone%252Fs%252Ffile%253Fid%3Dba62%252FPLOSOne_formatting_sample_title_authors_affiliations.pdf%2F1%2F010f019f1bc4a654-a34a84a7-1535-49a9-8388-3d7f80682798-000000%2FxFWsl7_aOLPJf7P2LhtYyR9T0Y58p-yswydZS4fh1UU%3D258&data=05%7C02%7Csd%40mrc.soton.ac.uk%7Ca6e94d276ea9414ace5d08ded722aa5d%7C4a5378f929f44d3ebe89669d03ada9d8%7C0%7C0%7C639184741380451302%7CUnknown%7CTWFpbGZsb3d8eyJFbXB0eU1hcGkiOnRydWUsIlYiOiIwLjAuMDAwMCIsIlAiOiJXaW4zMiIsIkFOIjoiTWFpbCIsIldUIjoyfQ%3D%3D%7C0%7C%7C%7C&sdata=2YedvBazZx%2BCPu0FCSSF2R5f%2BN34NcbOSTxI98JwnBo%3D&reserved=0)

Thank you. We have modified the manuscript accordingly.

2. Your ethics statement should only appear in the Methods section of your manuscript. If your ethics statement is written in any section besides the Methods, please move it to the Methods section and delete it from any other section. Please ensure that your ethics statement is included in your manuscript, as the ethics statement entered into the online submission form will not be published alongside your manuscript.

Thank you. This has been actioned.

3. In the online submission form, you indicated that the datasets used for this analysis are available on reasonable request from the MRC Lifecourse Epidemiology Centre by contacting the corresponding author: [sd@mrc.soton.ac.uk](mailto:sd@mrc.soton.ac.uk).

All PLOS journals now require all data underlying the findings described in their manuscript to be freely available to other researchers, either 1. In a public repository, 2. Within the manuscript itself, or 3. Uploaded as supplementary information.
This policy applies to all data except where public deposition would breach compliance with the protocol approved by your research ethics board. If your data cannot be made publicly available for ethical or legal reasons (e.g., public availability would compromise patient privacy), please explain your reasons on resubmission and your exemption request will be escalated for approval.

Thank you, this was an oversight. At the time of participation, written consent from participants was obtained on the basis that the data would be accessible only to the research team.

The exact wording was as below:

*“I understand that all information collected about me during my participation in this study will be stored in locked cabinets in a secure building or on a password protected computer, and that this information will be available only to the small study team, and used only for the purpose of this study. All files containing identifying information (names and addresses) will be kept separate from those containing other information about me, which will be identified only by a coded serial number. No information from the study will be published in a form that could lead to the identification of individuals.”*

This undertaking was part of the protocol that was approved by the National Health Service (NHS) Research Ethics Committee North West-Liverpool East. Thus, we are unable to make the dataset publicly available, and to do so would compromise patient privacy. We have amended the “Data availability” section accordingly.

4. Thank you for stating the following financial disclosure:
For the purpose of open access, the author has applied for a CC BY public copyright licence. The HEAF study is funded by the Medical Research Council programme grant (MC_UU_12011/5), and by grant awards from Versus Arthritis (formerly Arthritis Research UK) (22090); and the Economic and Social Research Council and Medical Research Council jointly (ES/L002663/1).

Please state what role the funders took in the study. If the funders had no role, please state: "The funders had no role in study design, data collection and analysis, decision to publish, or preparation of the manuscript."
If this statement is not correct you must amend it as needed.
Please include this amended Role of Funder statement in your cover letter; we will change the online submission form on your behalf.

Thank you. This has been actioned.

5. Please update your submission to use the PLOS LaTeX template. The template and more information on our requirements for LaTeX submissions can be found at http://journals.plos.org/plosone/s/latex.

6. If the reviewer comments include a recommendation to cite specific previously published works, please review and evaluate these publications to determine whether they are relevant and should be cited. There is no requirement to cite these works unless the editor has indicated otherwise.

Thank you for your comment. We have taken on board the reviewers’ suggestion and have expanded the literature cited.

Reviewers' comments:

Reviewer's Responses to Questions

**Comments to the Author**

1. Is the manuscript technically sound, and do the data support the conclusions?

The manuscript must describe a technically sound piece of scientific research with data that supports the conclusions. Experiments must have been conducted rigorously, with appropriate controls, replication, and sample sizes. The conclusions must be drawn appropriately based on the data presented.

Reviewer #1: Yes

Reviewer #2: Partly

Reviewer #3: Yes

2. Has the statistical analysis been performed appropriately and rigorously?

Reviewer #1: Yes

Reviewer #2: No

Reviewer #3: Yes

3. Have the authors made all data underlying the findings in their manuscript fully available?

The [PLOS Data policy](https://eur03.safelinks.protection.outlook.com/?url=http%3A%2F%2Ftrack.editorialmanager.com%2FCL0%2Fhttp%3A%252F%252Fwww.plosone.org%252Fstatic%252Fpolicies.action%2523sharing%2F1%2F010f019f1bc4a654-a34a84a7-1535-49a9-8388-3d7f80682798-000000%2FOkNoiX7LaY6vGPU5DDImEu-ZzwQsUQ8rdbpxjptF1Ns%3D258&data=05%7C02%7Csd%40mrc.soton.ac.uk%7Ca6e94d276ea9414ace5d08ded722aa5d%7C4a5378f929f44d3ebe89669d03ada9d8%7C0%7C0%7C639184741380487460%7CUnknown%7CTWFpbGZsb3d8eyJFbXB0eU1hcGkiOnRydWUsIlYiOiIwLjAuMDAwMCIsIlAiOiJXaW4zMiIsIkFOIjoiTWFpbCIsIldUIjoyfQ%3D%3D%7C0%7C%7C%7C&sdata=lP2cQRfjakuE%2FNBx0vrRzCiwwLK9MeBbsNBYoXR%2Fq7E%3D&reserved=0) requires authors to make all data underlying the findings described in their manuscript fully available without restriction, with rare exception (please refer to the Data Availability Statement in the manuscript PDF file). The data should be provided as part of the manuscript or its supporting information, or deposited to a public repository. For example, in addition to summary statistics, the data points behind means, medians and variance measures should be available. If there are restrictions on publicly sharing data—e.g. participant privacy or use of data from a third party—those must be specified.

Reviewer #1: Yes

Reviewer #2: No

Reviewer #3: Yes

4. Is the manuscript presented in an intelligible fashion and written in standard English?

PLOS ONE does not copyedit accepted manuscripts, so the language in submitted articles must be clear, correct, and unambiguous. Any typographical or grammatical errors should be corrected at revision, so please note any specific errors here.

Reviewer #1: Yes

Reviewer #2: Yes

Reviewer #3: Yes

5. Review Comments to the Author

Please use the space provided to explain your answers to the questions above. You may also include additional comments for the author, including concerns about dual publication, research ethics, or publication ethics. (Please upload your review as an attachment if it exceeds 20,000 characters)

Reviewer #1: Title:
- According to the proposed objectives and method, it is better to convey the title with a measurable format (e.g., the association of risk factors with early long-term economic inactivity in older workers).

Thank you for the suggestion. We have changed the title to *“Associations of sociodemographic, health, and work-related risk factors with early long-term economic inactivity in older workers: findings from the Health and Employment After Fifty study”*

Abstract:
- For a better understanding of the readers, please show the effect of the Job dissatisfaction, low physical work demands and slow walking speed, numerically in the result part of this section.

Thank you. This has been amended.

Keywords:
- OK
Introduction:
- It seems to be necessary to define the necessity and novelty and expected applied use of the results of the study explicitly and in more detail in this section.

Thank you. The introduction has been expanded to explain why the study was needed and where its results were expected to find application.

Method:
- In contrast to refereeing this section to another paper, it is necessary to define the method some how in more detail in terms of the following issues:
o Inclusion and exclusion criteria of the recruited participants.
o All of the proposed independent variables considered as probable risk factors of the outcome especially in terms of their measurements and the time of their measuring during the study. This may include the reliability measures as well.
o How the participants were followed up.
o How the loss to follow up has been addressed.

Thank you. The original plan was for the two papers to be published in the same journal, and we therefore sought to avoid unnecessary duplications across the manuscripts. However, as the companion paper has since been published in *BMJ Open*, we have expanded the Methods in the present manuscript to ensure it is fully self-contained and can stand independently.

Results:
- The levels of a number of variables such as housing tenure, contribution to family income, financial position, and … are not addressed in the method section (tables 2-4).

Thank you. We have expanded the methods section and added a supplementary table (S1 Table) with more details on the variables included in the analysis.

Discussion:
- OK.
References:
- OK.

Reviewer #2: This manuscript examines determinants of early economic inactivity among older workers using data from the Health and Employment After Fifty (HEAF) study. The topic is important from both a policy and public health perspective, given increasing concerns regarding labour market participation in later working life and the sustainability of extending working lives. The study benefits from the use of a rich and a relatively uncommon dataset with a broad range of exposures, and the analyses are generally clearly presented and employ largely appropriate statistical methods.

Thank you for your positive feedback on our manuscript.

However, I have several substantive concerns that limit my confidence in the interpretation and contribution of the findings. These relate primarily to: (1) the absence of a meaningful engagement with the existing literature, (2) the conceptualisation of economic inactivity as a highly heterogeneous outcome, and (3) concerns regarding sample representativeness, attrition, and missing data handling.

Major Comments
1. Insufficient Background and Lack of Literature Review
The manuscript contains very little engagement with the existing scientific literature. The introduction does not adequately establish what is already known about the determinants of economic inactivity, retirement, or labour market withdrawal in later life, making it difficult to assess the novelty of the research question or the contribution of the study findings.
Only ten references are included, four of which relate directly to the HEAF cohort. Notably, there is almost no citation of the substantial academic literature (outside of this study) examining retirement transitions, labour market exit, or economic inactivity among older adults. As a result, the study lacks any theoretical or empirical framework.
The authors should expand the introduction and discussion to situate their work within the broader literature. For example a few relevant examples for retirement might include
• Stiemke, P., & Hess, M. (2022). Determinants of (in-)voluntary retirement: A systematic literature review. Journal of European Social Policy, 32(3), 348–358.
• Topa, G., Depolo, M., & Alcover, C. M. (2018). Early retirement: a meta-analysis of its antecedent and subsequent correlates. Frontiers in Psychology, 8, 2157.
• Pilipiec, P., Groot, W., & Pavlova, M. (2022). The analysis of predictors of retirement preferences over time. Journal of Population Ageing, 15(2), 425–452.
• Wilson, D. M., et al. (2020). Identifying contemporary early retirement factors and strategies to encourage and enable longer working lives: A scoping review. International Journal of Older People Nursing, 15(3), e12313.
Without such contextualisation, it is difficult to understand whether the findings confirm, contradict, or extend previous evidence.

Thank you. We agree with the reviewer that the introduction did not cite all the relevant literature in this area. We have modified the introduction to make clearer the context of our investigation, summarising the available evidence on determinants of early retirement or exit from paid employment.

Relatedly, the selection of antecedent variables appears to be based largely on another manuscript by the authors that is currently under review and ‘All had been identified at the time when the study was being set up as plausible determinants of exit from paid work (line 54). How? Where from? The other study under review is not yet publicly available, readers cannot evaluate the rationale for variable selection. Predictor selection should be justified through reference to the established literature. Also, the specification of independent variables (line 58) should be included in the supplementary materials.

Thank you. Our original idea was for the two papers to be published together as we see them as complementing one another. However, the paper on risk factors of high annual sickness absence has since been published elsewhere. We have expanded the methods section and, as suggested by the reviewer, we have added a Supplementary table (S1 Table) reporting how questions were asked, and how risk factors were derived for the analysis.

2. Economic Inactivity is a Highly Heterogeneous Outcome
A central concern is the treatment of "economic inactivity" as a single outcome category. This combines individuals who may have exited the labour market for very different reasons, including retirement, long-term sickness or disability, caring responsibilities, and long-term unemployment.
These pathways likely have distinct determinants and may represent fundamentally different processes. Combining them into a single outcome risks obscuring important heterogeneity and may complicate interpretation of the reported associations.
At minimum, the authors should provide descriptive information on the distribution of types of economic inactivity among participants who experienced the outcome (if possible at each interval). Authors should provide a stronger justification for treating these groups as equivalent.

Thank you for this comment. We agree with the reviewer that different factors may influence long-term exit from paid work at older ages, but we think it would be simplistic to assume that in each individual, the transition can be ascribed unambiguously to a single reason. It is eminently plausible that for some people, several factors act in combination. Therefore, we think it is preferable to base our outcome on economic inactivity overall, and not to attempt any sub-division of the sort that the reviewer proposes. The relative importance of influences such as sickness or caring responsibilities can then be assessed through analysis.

As now reported in the Methods section, where a participant left a job, we did ask whether that was at least partly for health reasons. We have added a note in the Results section that of the 682 individuals who experienced ELTEI during follow-up, 208 (30.5%) reported that the job loss was at least partly related to health, while the remaining 474 (60.5%) said that it was not.

3. Representativeness, Attrition, and Selection Bias
The manuscript suggests that the study sample is representative, including the statement in the strengths and limitations section that the cohort "should be representative." This claim is not sufficiently supported.
The HEAF cohort was not fully representative of the target population at recruitment (as stated in the cohort profile), and the analytic sample used in the present study has been further reduced through attrition and inclusion criteria. Specifically, of the sample of interest approximately one quarter (1,058 + 51 / (3450+1,109) appears to have been lost prior to analysis. Such losses are unlikely to be random, particularly given the strong links between health, employment status, and study participation.
The discussion includes some consideration of representativeness (lines 199–205), but there is little consideration of non-response related to poor health, socioeconomic disadvantage, or labour market detachment. These factors may have important implications for both external validity and internal validity.
Furthermore, no survey weights or other adjustments appear to have been applied. In the absence of formal assessment of representativeness or weighting procedures, claims that the analytical sample is representative should be removed or substantially qualified.
The authors should provide:
• Characteristics of included versus excluded participants where possible.
• Discussion of the potential for selection bias arising from non-response and attrition.
• Justification for any claims regarding representativeness.

Thank you for this comment. The strengths and limitations section should have referred to “People invited to take part in the study …” rather than “Participants invited to take part in the study ….” and we have made this correction. Also, it includes the qualifier “broadly” before “representative”, which the reviewer seems to have overlooked. In full, therefore, it now reads “People invited to take part in the study should have been broadly representative of the national population”.

Its justification stems from the geographical spread of contributing general practices (as outlined in the first paragraph of the Methods section), the fact that in Britain almost everyone is registered with a general practice (meaning that their lists give almost complete coverage of the population), and the only exclusions from eligibility being for terminal illness or recent bereavement (again as mentioned in paragraph 1 of the Methods section).

More important is the potential for bias from incomplete participation of those originally invited to join the study, and from incomplete follow-up of participants who were at risk of ELTEI when they completed the baseline questionnaire. We have no information about the characteristics of people who declined to join the study other than that they met the age criteria for inclusion. However, in what is now paragraph 3 of the Discussion, we acknowledge that “The incomplete participation of those invited to join the study (21%) leaves open the possibility that the final study sample was in some respects importantly unrepresentative of the national population. For example, there may have been over-representation of people with higher levels of education and employed in non-manual jobs.”

Regarding incomplete follow-up, we have now added further information in paragraph 1 of the Results section and S2 Table comparing the prevalence of baseline characteristics of participants who initially were at risk of ELTEI according to the completeness of follow-up information. Among the latter, participants who provided at least some usable follow-up (and therefore contributed to the assessment of risk factors) tended to have higher educational attainment, report better self-rated health, and be less likely currently to smoke than those who provided no usable follow-up data. They were also less likely to reside in more deprived areas. However, among participants who contributed to the assessment of risk factors, there were no notable differences in baseline characteristics between those who returned all required questionnaires and those for whom follow-up was censored because of failure to return a questionnaire.

There is a possibility that drop-out from follow-up was related to recent onset of illness not captured in earlier questionnaires, but we have no way of knowing to what extent such illness would make people more or less likely to return a questionnaire.

The potential for selection bias will depend on whether people who were excluded from, or contributed less to, analysis because they failed to return questionnaires differed importantly from those who contributed maximally in regard to the associations under investigation. In other words, what matters ultimately is the representativeness of participants specifically with regard to the associations under investigation. As we say in paragraph 3 of the Discussion, despite observed or likely differences in some characteristics, “we have no reason to expect that in participants who failed to provide maximal information, associations of ELTEI with risk factors would have been substantially different from those that we found using the data that were available”.

In summary, we have added further information comparing known characteristics of participants with full follow-up and those excluded from analysis because of incomplete follow-up information. We have expanded our discussion of the potential for bias from non-response and attrition. We do not have data with which to assess the potential for such bias, but we stand by our position that we have no reason to expect that in participants who failed to provide maximal information, associations of ELTEI with risk factors would have been substantially different from those reported.

4. Missing Data Handling
Missing data on items were handled by creating missing-value categories for covariates. While the proportion of missing data appears relatively very small, this approach has well-recognised limitations and may introduce bias into estimated associations. Dor example in the mutually adjusted models missingness was related to job satisfaction and expected income.
The economic inactivity data was ‘imputed’ using a single date following a set of rules established by the authors. This was the case for ‘469’ (line 94) participants – please could you state the number of times this was done. Also, this group seemed to be younger (less likely to be over 60). Using such an approach treats the value as a known, rather than an informed guesstimate. If used, could you call it ‘rule-based single imputation’ rather than imputed which is slightly misleading and outline the limitations.
Given the richness of the dataset, multiple imputation would appear both feasible and preferable for dealing with item non-response and attrition (to the sample of interest). Ideally, the analysis should be conducted with multiply imputed data or a sensitivity analysis comparing complete-case, missing-indicator, and multiple-imputation approaches should be presented to assess the robustness of findings. At minimum, the authors should justify their chosen approach and discuss its limitations further.

Thank you. It appears that the reviewer is conflating two different issues: one is that of “imputing” some dates of leaving or starting jobs and the other is the use of missing value categories for certain covariates.

To address the first point, a total of 469 individuals had either the date of leaving a previous job or the date of starting a new job missing. The current dataset structure does not permit identification of the total number of missing date occurrences; only the number of individuals with at least one missing date can be determined. As these dates were required to define the study outcome, we imputed the missing dates according to the rules described in the Methods section. However, no imputation was undertaken when both dates (job left and next job started) were missing. To assess the impact of this approach, we conducted a sensitivity analysis, excluding these 469 individuals. The results were materially unchanged, supporting the validity of our approach. Following the reviewer’s suggestion, we have changed the term to “rule-based single imputation”.

The second issue has to do with using a missing-value category for some covariates. We chose this approach as the proportion of missing values was small across each covariate, allowing us to retain the maximum sample size while avoiding the assumptions underlying multiple imputation. We now acknowledge in the Discussion that this approach may have introduced bias if case missingness was informative.

Additional Comments
5. Choice of Outcome Category
Line 187 indicates that the study used as justification for the outcome category - economic inactivity of 12 months or longer before SPa as similar to other studies. Reference Shaw et al. (2025) – this studies’ time period was very likely determined by interview timing during the COVID-19 pandemic and appears to be a pragmatic rather than scientifically motivated decision. The authors should provide a stronger justification for this choice.

Thank you. In this circumstance, any chosen cut-point in time will be to some extent arbitrary. Considerations include practicability given the data that are available (we had to be able to ascertain the outcome from the annual follow-up questionnaires), relevance to the purpose of the study (it needed to reflect a substantial impact on productivity) and (desirable but not essential) consistency with definitions used elsewhere (to facilitate comparison of findings). We have added a new paragraph 2 to the Discussion in which we expand on our rationale for the specified outcome.

Also, Line 195 the authors say, ‘the outcome that we chose was both more objective than self-reported retirement status, and a better measure of long-term impact on personal productivity’. How do they know this? Did they conduct a sensitivity analysis?

The assertion is a logical imputation and there is no need for a sensitivity analysis. Important loss of productivity will depend on the individual not being in paid work for a substantial period, regardless of whether he/she intends to return to paid work at some stage in the future. That a person is actively seeking work or intends to return to work at some stage in the future (and is therefore not classified as retired) does not reduce the impact on productivity from his not being at work.

We have tried to make our reasoning clearer in the new paragraph 2 of the Discussion.

6. Mutually adjusted Modelling Strategy
Several aspects of the mutually adjusted analyses require clarification:
• Why was educational attainment excluded from the mutually adjusted model? It was a determinant for both men and women?

• Marital status appears to have been excluded for men because it was not statistically significant in descriptive analyses. However, marital status is a well-established determinant of retirement behaviour, caregiving responsibilities, health trajectories, and labour market participation. Variable inclusion in multivariable models should generally also be informed by theory and prior evidence rather than solely by statistical significance in univariable analyses. Consider retaining marital status for both men and women.

Thank you for these suggestions. This was an oversight and we have amended the list of risk factors included in the final model. Estimates reporting in Table 5 have been updated accordingly.

7. Potential Age-Stratified Analyses
Determinants of economic inactivity are likely to differ substantially across age bands within the “older worker” category. Some consideration should be given to motivations for inactivity nearer SPa (noted that the authors remove everyone 2 years prior to SPa) may differ markedly from those occurring earlier in later working life. Perhaps, sample permitting the manuscript would benefit from further exploration of heterogeneity by splitting the age group (at least as a sensitivity analysis) by age first left. Stratified analyses by age could strengthen the findings and improve interpretability.

Thank you for this comment. As the median interval between ELTEI and SPA was 3.2 years, we explored potential heterogeneity in the effect of age by stratifying the mutually adjusted analyses presented in Table 5 according to proximity to SPA at the start of the interval. Specifically, participants were classified as being either within 4 years of SPA or 4 or more years from SPA. Due to sparse data in some categories, several of the confidence intervals are relatively wide. When this is taken into account, the estimates do not point clearly to heterogeneity. The results of the stratified analyses are shown in the table below (age at baseline is omitted because by definition, proximity to SPA is directly determined by age at baseline).

|  | **Men** | | **Women** | |
| --- | --- | --- | --- | --- |
|  | **HR (95% CI)** | | **HR (95% CI)** | |
|  | Less than 4 years from SPA (n=238) | 4+ years from SPA (n=1438) | Less than 4 years from SPA (n=85) | 4+ years from SPA (n=1689) |
| Level of education |  |  |  |  |
| No qualification/school only | Ref | Ref | Ref | Ref |
| Vocational training certificate | 0.57 (0.28,1.17) | 0.96 (0.66,1.40) | 1.91 (0.85,4.33) | 0.91 (0.68,1.21) |
| University degree or higher | 1.28 (0.72,2.30) | 1.35 (0.94,1.93) | 1.81 (0.67,4.84) | 1.19 (0.91,1.57) |
| Marital status |  |  |  |  |
| Married/civil partnership/co-habiting | Ref | Ref | Ref | Ref |
| Single/widowed/divorced | 1.36 (0.74,2.51) | 1.30 (0.96,1.76) | 0.34 (0.13,0.88) | 0.73 (0.56,0.96) |
| Missing | - | - | 2.71 (0.28,25.90) | 0.69 (0.22,2.17) |
| Contribution to family income (baseline) |  |  |  |  |
| Half or more | Ref | Ref | Ref | Ref |
| Between a quarter and a half | 0.83 (0.39,1.78) | 1.66 (1.17,2.35) | 1.09 (0.45,2.66) | 0.99 (0.75,1.30) |
| Less than a quarter | 1.98 (0.69,5.64) | 2.06 (1.22,3.48) | 2.30 (0.92,5.77) | 1.60 (1.19,2.16) |
| Missing | - | 1.78 (0.41,7.77) | 1.14 (0.11,12.35) | 0.56 (0.15,2.14) |
| Housing tenure (baseline) |  |  |  |  |
| Rented | Ref | Ref | Ref | Ref |
| Mortgaged | 1.44 (0.47,4.47) | 0.82 (0.47,1.44) | 0.33 (0.07,1.53) | 1.37 (0.83,2.27) |
| Owned outright or rent-free | 2.06 (0.70,6.11) | 1.61 (0.94,2.76) | 0.62 (0.15,2.55) | 1.81 (1.10,2.97) |
| Missing | - | 0.17 (0.01,2.14) | 1.42 (0.04,49.58) | 2.46 (0.54,11.13) |
| Receive or expect private/employer pension (baseline) |  |  |  |  |
| No | Ref | Ref | Ref | Ref |
| Expect to receive at/after SPA | 0.97 (0.31,3.04) | 0.83 (0.37,1.86) | 1.14 (0.33,3.83) | 0.64 (0.42,0.97) |
| Expect to receive before SPA | 1.32 (0.40,4.34) | 2.49 (1.33,4.66) | 4.40 (1.43,13.47) | 1.04 (0.75,1.43) |
| Already receive | 1.09 (0.36,3.29) | 1.96 (1.01,3.80) | 1.59 (0.56,4.51) | 1.04 (0.72,1.50) |
| Missing | - | 3.25 (0.41,25.68) | - | - |
| Expected total income after retirement compared to that at baseline (baseline) |  |  |  |  |
| Less than half | Ref | Ref | Ref | Ref |
| Between a quarter and a half | 2.83 (1.06,7.58) | 2.04 (1.31,3.17) | 0.72 (0.31,1.66) | 1.18 (0.90,1.54) |
| Half or more | 3.54 (1.30,9.59) | 1.98 (1.24,3.16) | 0.75 (0.27,2.05) | 1.49 (1.10,2.02) |
| Missing | 2.18 (0.42,11.43) | 2.84 (1.38,5.86) | 1.35 (0.36,5.11) | 1.32 (0.81,2.16) |
| Other unpaid work (time-varying) |  |  |  |  |
| No | Ref | Ref | Ref | Ref |
| Yes | 0.60 (0.27,1.35) | 1.32 (0.93,1.89) | 1.29 (0.56,2.98) | 1.20 (0.90,1.61) |
| Employment status |  |  |  |  |
| Self-employed | Ref | Ref | Ref | Ref |
| Employed | 1.72 (0.88,3.39) | 1.52 (1.03,2.23) | 1.36 (0.45,4.11) | 1.61 (1.10,2.37) |
| Physical demands of work |  |  |  |  |
| Low | Ref | Ref | Ref | Ref |
| Intermediate | 0.98 (0.56,1.70) | 0.97 (0.70,1.33) | 1.07 (0.53,2.19) | 0.92 (0.72,1.17) |
| Heavy | 0.54 (0.27,1.09) | 0.93 (0.64,1.35) | 1.19 (0.44,3.20) | 0.62 (0.42,0.91) |
| Missing | - | 6.29 (1.92,20.55) | - | 1.49 (0.41,5.44) |
| Easily coping with both physical and mental demands of the job |  |  |  |  |
| Yes |  |  | Ref | Ref |
| No |  |  | 1.79 (0.83,3.87) | 1.56 (1.22,1.99) |
| Missing |  |  | - | 3.73 (0.84,16.61) |
| Overall job satisfaction |  |  |  |  |
| Satisfied | Ref | Ref | Ref | Ref |
| Dissatisfied | 1.15 (0.43,3.04) | 1.89 (1.25,2.88) | 2.12 (0.73,6.17) | 1.29 (0.89,1.88) |
| Missing | 3.92 (0.77,19.96) | 1.19 (0.42,3.35) | - | 2.33 (0.61,8.84) |
| Disabling pain in back, neck or limbs in past 12 months |  |  |  |  |
| No |  |  | Ref | Ref |
| Yes |  |  | 1.05 (0.48,2.27) | 1.28 (0.98,1.67) |
| Missing |  |  | - | 1.07 (0.15,7.76) |
| Depression |  |  |  |  |
| No |  |  | Ref | Ref |
| Yes |  |  | 0.94 (0.43,2.05) | 1.06 (0.81,1.37) |
| Missing |  |  | - | - |
| Walking speed |  |  |  |  |
| Normal/Fast | Ref | Ref | Ref | Ref |
| Very slow/Unable to walk | 1.67 (0.38,7.35) | 3.94 (2.24,6.92) | 1.34 (0.32,5.64) | 1.75 (1.13,2.72) |
| Missing | 1.35 (0.17,10.92) | - | 1.84 (0.06,54.16) | - |

8. Data Availability Statement
The manuscript states:
"The datasets used for this analysis are available on reasonable request from the MRC Lifecourse Epidemiology Centre by contacting the corresponding author."
This statement does not comply with PLOS ONE data availability requirements.

Thank you for this comment. We have amended the “Data Availability Statement” to reflect the consent from participants which makes data only available to the research team.

9. Writing and Presentation
The manuscript is generally understandable and reasonably well organised. However, there are several instances of repetitive phrasing, missing words, and minor grammatical issues. A careful editorial review would improve readability.

Thanks for the comment. We try to avoid repetitive phrasing except where it is necessary for clarity. We have not identified any unequivocal grammatical errors, but would be happy to respond on specific points of grammar if highlighted.

10. Also:
- line 44: ‘was first onset’ –needs clarifying - over the age of 50?

As in any cohort study, the outcome had to occur during the follow-up period, and since all participants were aged 50 years or older at baseline, the outcome had to occur over the age 50. In theory, however, a person could have experienced onset of ELTEI at age 51, returned to work at age 54, entered the study at age 56 and then experienced a second onset of ELTEI at age 58. The second onset would meet the criteria for the outcome. However, it would not be the person’s first onset of ELTEI over the age of 50.

We suggest that any attempt to expand the current wording (which is accurate) might cause unwarranted confusion.

- line 64: ‘ Analysis was based on participants who, at the time of recruitment, were in a paid job…’ the study will possibly not include those temporarily LT sick at the time of interview, nor those, especially women coming back into the workplace. Should be mentioned as a limitation.

Thanks for the opportunity to clarify. Participants on sick leave, whether employed or self-employed, were classed as being in paid work and therefore were included in the analytic sample. We have amended the text:

*“Analysis was based on participants who, at the time of recruitment, were in a paid job (including those on sick leave), would not reach their SPA for more than two years, and who subsequently completed at least the first two follow-up questionnaires.”*

- Do you have information on the types of pensions participants had i.e. those with a Defined Benefit pension, and the type of work they did (e.g Standard Occupational Classification), public or private sector which are determinants of leaving the workforce?

We do not have information on the types of pensions which participants received. We did collect information about jobs held at baseline and over the course of follow-up but could not reliably classify work as being in the public or private sector.

Overall Assessment
This study addresses an important topic and utilises a valuable longitudinal dataset. However, the manuscript currently lacks sufficient engagement with the existing literature, does not adequately address the heterogeneity of economic inactivity, and raises concerns regarding representativeness, attrition, and missing data handling. Addressing these issues would substantially strengthen the scientific contribution and improve confidence in the reported findings.

Reviewer #3: 1. Sex-stratified analyses are insufficiently justified. Results are presented separately for men and women, but the manuscript does not state whether formal sex-by-exposure interaction terms were tested before stratification. The authors should either report interaction tests or justify sex stratification as an a priori analytic decision.

Thank you for the comment. We have added a sentence specifying that the choice of sex stratification was done a priori and was based on the well-known differences in employment circumstances and pathways out of employment between men and women. Please see the new text below.

*“This strategy was specified a priori because work circumstances and pathways out of employment are known to differ by sex”*

2 Potential selection and attrition bias. The analytic sample excludes participants not in paid work at baseline and those without the first two follow-up questionnaires, which may create a selected healthier or more stable working population. The authors should discuss how this may bias the observed associations.

Our consideration of this and other possible sources of bias is dealt with at length in our response to Reviewer #2.

3. Data-driven model selection. The multivariable model appears to include variables based on age-adjusted statistical significance or confidence interval criteria. This approach may omit important confounders and overemphasize chance findings; a theory-based confounder selection strategy would be preferable.

The selection of variables included in our multivariable models was not data-driven alone. The potential risk factors were identified a priori when the HEAF study was set up, based on existing evidence and plausible pathways through which demographic, health, employment, financial, and personal factors might influence long-term worklessness. The analysis for Table 5 was intended to identify those that were most important, and as reported in paragraph 2 of the section on methods of statistical analysis, we checked for the possibility that an important risk factor might have been obscured by negative confounding.

4 Unclear handling of time-varying exposures. Several predictors vary over time, but the manuscript uses the most recent known value at the start of each interval. This may misclassify exposure status during follow-up and should be more clearly justified.

Thank you for this comment. In the manuscript we have clearly specified which covariates were treated as fixed (as reported as baseline) and which as time-varying. All variables related to health and work characteristics were liable to change over follow-up, assessed repeatedly and analysed as time-varying. For-time varying, covariates, we used the most recently available value, which was from the start of the follow-up interval under consideration. This approach ensured appropriate temporal ordering between exposure and outcome, but there may sometimes have been changes in a risk factor between the start of an interval and an onset of ELTEI later in the same interval. This is perhaps more likely to have occurred for variables related to health than to employment, and the resultant misclassification may have attenuated some associations. We have added brief consideration of it in paragraph 5 of the Discussion.

5. Outcome timing may be imprecise. Extended labour market exit is defined as being continuously out of paid work for at least 12 months, but annual questionnaires and imputed dates may limit precision in identifying onset. The authors should clarify how timing uncertainty was handled.

Timing of the outcome was determined by the reported date on which the last paid job was left before the period of economic inactivity. This required recall over a period of up to 12 months, but the exact date would be important only if the individual returned to paid work very close to 12 months later. There would then be a question as to whether the period of economic inactivity reliably did or did not exceed 12 months. In practice, borderline cases of this sort did not occur, and the bigger potential for error was from missing information on dates of leaving or starting jobs. This was dealt with by rule-based single imputation, and the potential for consequent bias was checked through a sensitivity analysis which excluded intervals with information based on imputed dates.

6. Limited discussion of missing data. Missing values are included as categories in some analyses,but no clear multiple imputation or sensitivity analysis strategy is described. This may bias estimates if missingness is related to employment exit or key exposures.

Thank you. We chose this approach as the prevalence of missing values was small across each covariate, and it allowed us to retain maximum sample size while avoiding the assumptions underlying multiple imputation. We have acknowledged that this approach may introduce bias in case missingness is informative in the Discussion section.

*“We acknowledge our use of missing-value categories as a potential limitation. We chose this approach because the proportion of missing data across variables was small, and it allowed us to retain a maximum sample size without relying on the assumptions required for multiple imputation. It is possible that the missing-indicator approach introduced bias if missingness itself were informative, but given the low prevalence of missing data, any such bias is likely to have been small.”*

7. Risk of multicollinearity and overadjustment. Financial difficulty, employment status,job insecurity, and work-related factors are likely correlated, and some may lie on causal pathways. The authors should clarify which variables are confounders versus mediators.

Thank you. We have added a sentence clarifying that we have assessed multicollinearity using variance inflation factors (VIFs) calculated from a linear regression model containing the same predictor variables. No evidence of problematic collinearity was observed.

8. Interpretation may be too causal. The conclusions emphasize financial and employment-related drivers of exit, but the observational design limits causal inference.The wording should be more cautious.

Thank you. The wording that we use in our concluding paragraph is “Economic inactivity seems to be driven predominantly by considerations of personal finance and affordability and is more common in people with higher levels of education and in occupations that are less demanding physically. In contrast, high sickness absence occurs most frequently in those from poorer social circumstances with less education, poor personal health, and jobs that are more demanding physically. In other words, the people whom increases in SPA most often oblige to work to older ages tend to be those who will find it most difficult to do so. Because of their lower average level of education, they will often also have started productive employment at a younger age.” The only part of this which might be taken to imply causation is the term “seems to be driven” but that is hedged by the “seems to be”. Moreover, mechanistically it is highly plausible that personal financial security would influence decisions to exit paid work early, which was why we included in our investigation.

For consistency, we have added the words “seems to be” to the conclusions in the Abstract.

9Sparse data in some subgroups. Some sex-specific estimates have wide confidence intervals, suggesting limited precision. These findings should be interpreted cautiously.

Thank you. We have adjusted the language to avoid too much emphasis when estimates are based on sparse data.

10. Proportional hazards or model assumptions are not discussed. The authors should state whether assumptions of the discrete-time Cox approach were checked.

Thank you. This was an oversight. We have added to the Statistical analysis section

*“Tests of the proportional hazards assumption in the final models were based on Schoenfeld residuals.”*

11. Results are somewhat fragmented. Age-adjusted and mutually adjusted findings are spread across tables, making interpretation difficult. A clearer presentation of the main model would improve readability.

Our method of presenting results was carefully considered, with the aim of clarity and efficiency. A single table including age-adjusted results for all variables and mutually adjusted results for a selected subset of variables would to our mind be extremely unwieldy. We acknowledge that this is a matter of subjective personal preferences, but we prefer the approach that we have adopted, which is similar to that used in many previous papers by ourselves and others.
