# Supplementary material for: Associations of sociodemographic, health, and work-related factors with early long-term economic inactivity in older workers: Findings from the Health and Employment After Fifty study
Source: PLoS One. 2026 Oct 6;21(10):e0359865. doi: 10.1371/journal.pone.0359865 (PMC13641384; doi:10.1371/journal.pone.0359865)
Supplement: Peer review history

# Peer review: Decision Letter 0

**DOI:** 10.1371/journal.pone.0359865.r001

**Authors:** Hamid Reza Baradaran

30 Jun 2026

Dear Dr. D'Angelo,

Thank you for submitting your manuscript to PLOS ONE. After careful consideration, we feel that it has merit but does not fully meet PLOS ONE’s publication criteria as it currently stands. Therefore, we invite you to submit a revised version of the manuscript that addresses the points raised during the review process.

This an interesting study with appropriate design but needs a strong and rubout  analysis , it is necessary to  revise some aspect of analysis as revivers  have mentioned in their comments .

==================================

Please submit your revised manuscript by Aug 29 2026 11:59PM. If you will need more time than this to complete your revisions, please reply to this message or contact the journal office at plosone@plos.org. When you're ready to submit your revision, log on to https://www.editorialmanager.com/pone/ and select the 'Submissions Needing Revision' folder to locate your manuscript file.

- A letter that responds to each point raised by the academic editor and reviewer(s). You should upload this letter as a separate file labeled 'Response to Reviewers'.
- A marked-up copy of your manuscript that highlights changes made to the original version. You should upload this as a separate file labeled 'Revised Manuscript with Track Changes'.
- An unmarked version of your revised paper without tracked changes. You should upload this as a separate file labeled 'Manuscript'.

If you would like to make changes to your financial disclosure, please include your updated statement in your cover letter. Guidelines for resubmitting your figure files are available below the reviewer comments at the end of this letter.

If applicable, we recommend that you deposit your laboratory protocols in protocols.io to enhance the reproducibility of your results. Protocols.io assigns your protocol its own identifier (DOI) so that it can be cited independently in the future. For instructions see: https://journals.plos.org/plosone/s/submission-guidelines#loc-laboratory-protocols. Additionally, PLOS ONE offers an option for publishing peer-reviewed Lab Protocol articles, which describe protocols hosted on protocols.io. Read more information on sharing protocols at https://plos.org/protocols?utm_medium=editorial-email&utm_source=authorletters&utm_campaign=protocols.

As the corresponding author, your ORCID iD is verified in the submission system and will appear in the published article. PLOS supports the use of ORCID, and we encourage all coauthors to register for an ORCID iD and use it as well. Please encourage your coauthors to verify their ORCID iD within the submission system before final acceptance, as unverified ORCID iDs will not appear in the published article. *Only*  the individual author can complete the verification step; PLOS staff *cannot*  verify ORCID iDs on behalf of authors.

We look forward to receiving your revised manuscript.

Kind regards,

Hamid Reza Baradaran, M.D., Ph.D.,

Academic Editor

PLOS One

Journal Requirements:

When submitting your revision, we need you to address these additional requirements.

1. Please ensure that your manuscript meets PLOS ONE's style requirements, including those for file naming. The PLOS ONE style templates can be found at

https://journals.plos.org/plosone/s/file?id=wjVg/PLOSOne_formatting_sample_main_body.pdf and https://journals.plos.org/plosone/s/file?id=ba62/PLOSOne_formatting_sample_title_authors_affiliations.pdf

2. Your ethics statement should only appear in the Methods section of your manuscript. If your ethics statement is written in any section besides the Methods, please move it to the Methods section and delete it from any other section. Please ensure that your ethics statement is included in your manuscript, as the ethics statement entered into the online submission form will not be published alongside your manuscript.

3. In the online submission form, you indicated that the datasets used for this analysis are available on reasonable request from the MRC Lifecourse Epidemiology Centre by contacting the corresponding author: sd@mrc.soton.ac.uk.

All PLOS journals now require all data underlying the findings described in their manuscript to be freely available to other researchers, either 1. In a public repository, 2. Within the manuscript itself, or 3. Uploaded as supplementary information.

This policy applies to all data except where public deposition would breach compliance with the protocol approved by your research ethics board. If your data cannot be made publicly available for ethical or legal reasons (e.g., public availability would compromise patient privacy), please explain your reasons on resubmission and your exemption request will be escalated for approval.

4. Thank you for stating the following financial disclosure:

For the purpose of open access, the author has applied for a CC BY public copyright licence. The HEAF study is funded by the Medical Research Council programme grant (MC_UU_12011/5), and by grant awards from Versus Arthritis (formerly Arthritis Research UK) (22090); and the Economic and Social Research Council and Medical Research Council jointly (ES/L002663/1).

Please state what role the funders took in the study. If the funders had no role, please state: "The funders had no role in study design, data collection and analysis, decision to publish, or preparation of the manuscript."

If this statement is not correct you must amend it as needed.

Please include this amended Role of Funder statement in your cover letter; we will change the online submission form on your behalf.

5. Please update your submission to use the PLOS LaTeX template. The template and more information on our requirements for LaTeX submissions can be found at http://journals.plos.org/plosone/s/latex.

6. If the reviewer comments include a recommendation to cite specific previously published works, please review and evaluate these publications to determine whether they are relevant and should be cited. There is no requirement to cite these works unless the editor has indicated otherwise.

Additional Editor Comments :

This an interesting study with appropriate design but needs a strong and rubout analysis , it is necessary to revise some aspect of analysis as revivers have mentioned in their comments .

[Note: HTML markup is below. Please do not edit.]

Reviewers' comments:

Reviewer's Responses to Questions

**Comments to the Author**

1. Is the manuscript technically sound, and do the data support the conclusions?

Reviewer #1: Yes

Reviewer #2: Partly

Reviewer #3: Yes

**********

2. Has the statistical analysis been performed appropriately and rigorously?

Reviewer #1: Yes

Reviewer #2: No

Reviewer #3: Yes

**********

3. Have the authors made all data underlying the findings in their manuscript fully available?

The PLOS Data policy

Reviewer #1: Yes

Reviewer #2: No

Reviewer #3: Yes

**********

4. Is the manuscript presented in an intelligible fashion and written in standard English?

Reviewer #1: Yes

Reviewer #2: Yes

Reviewer #3: Yes

**********

Reviewer #1: Title:

- According to the proposed objectives and method, it is better to convey the title with a measurable format (e.g., the association of risk factors with early long-term economic inactivity in older workers).

Abstract:

- For a better understanding of the readers, please show the effect of the Job dissatisfaction, low physical work demands and slow walking speed, numerically in the result part of this section.

Keywords:

- OK

Introduction:

- It seems to be necessary to define the necessity and novelty and expected applied use of the results of the study explicitly and in more detail in this section.

Method:

- In contrast to refereeing this section to another paper, it is necessary to define the method some how in more detail in terms of the following issues:

o Inclusion and exclusion criteria of the recruited participants.

o All of the proposed independent variables considered as probable risk factors of the outcome especially in terms of their measurements and the time of their measuring during the study. This may include the reliability measures as well.

o How the participants were followed up.

o How the loss to follow up has been addressed.

Results:

- The levels of a number of variables such as housing tenure, contribution to family income, financial position, and … are not addressed in the method section (tables 2-4).

Discussion:

- OK.

References:

- OK.

Reviewer #2: This manuscript examines determinants of early economic inactivity among older workers using data from the Health and Employment After Fifty (HEAF) study. The topic is important from both a policy and public health perspective, given increasing concerns regarding labour market participation in later working life and the sustainability of extending working lives. The study benefits from the use of a rich and a relatively uncommon dataset with a broad range of exposures, and the analyses are generally clearly presented and employ largely appropriate statistical methods.

However, I have several substantive concerns that limit my confidence in the interpretation and contribution of the findings. These relate primarily to: (1) the absence of a meaningful engagement with the existing literature, (2) the conceptualisation of economic inactivity as a highly heterogeneous outcome, and (3) concerns regarding sample representativeness, attrition, and missing data handling.

Major Comments

1. Insufficient Background and Lack of Literature Review

The manuscript contains very little engagement with the existing scientific literature. The introduction does not adequately establish what is already known about the determinants of economic inactivity, retirement, or labour market withdrawal in later life, making it difficult to assess the novelty of the research question or the contribution of the study findings.

Only ten references are included, four of which relate directly to the HEAF cohort. Notably, there is almost no citation of the substantial academic literature (outside of this study) examining retirement transitions, labour market exit, or economic inactivity among older adults. As a result, the study lacks any theoretical or empirical framework.

The authors should expand the introduction and discussion to situate their work within the broader literature. For example a few relevant examples for retirement might include

• Stiemke, P., & Hess, M. (2022). Determinants of (in-)voluntary retirement: A systematic literature review. Journal of European Social Policy, 32(3), 348–358.

• Topa, G., Depolo, M., & Alcover, C. M. (2018). Early retirement: a meta-analysis of its antecedent and subsequent correlates. Frontiers in Psychology, 8, 2157.

• Pilipiec, P., Groot, W., & Pavlova, M. (2022). The analysis of predictors of retirement preferences over time. Journal of Population Ageing, 15(2), 425–452.

• Wilson, D. M., et al. (2020). Identifying contemporary early retirement factors and strategies to encourage and enable longer working lives: A scoping review. International Journal of Older People Nursing, 15(3), e12313.

Without such contextualisation, it is difficult to understand whether the findings confirm, contradict, or extend previous evidence.

Relatedly, the selection of antecedent variables appears to be based largely on another manuscript by the authors that is currently under review and ‘All had been identified at the time when the study was being set up as plausible determinants of exit from paid work (line 54). How? Where from? The other study under review is not yet publicly available, readers cannot evaluate the rationale for variable selection. Predictor selection should be justified through reference to the established literature. Also, the specification of independent variables (line 58) should be included in the supplementary materials.

2. Economic Inactivity is a Highly Heterogeneous Outcome

A central concern is the treatment of "economic inactivity" as a single outcome category. This combines individuals who may have exited the labour market for very different reasons, including retirement, long-term sickness or disability, caring responsibilities, and long-term unemployment.

These pathways likely have distinct determinants and may represent fundamentally different processes. Combining them into a single outcome risks obscuring important heterogeneity and may complicate interpretation of the reported associations.

At minimum, the authors should provide descriptive information on the distribution of types of economic inactivity among participants who experienced the outcome (if possible at each interval). Authors should provide a stronger justification for treating these groups as equivalent.

3. Representativeness, Attrition, and Selection Bias

The manuscript suggests that the study sample is representative, including the statement in the strengths and limitations section that the cohort "should be representative." This claim is not sufficiently supported.

The HEAF cohort was not fully representative of the target population at recruitment (as stated in the cohort profile), and the analytic sample used in the present study has been further reduced through attrition and inclusion criteria. Specifically, of the sample of interest approximately one quarter (1,058 + 51 / (3450+1,109) appears to have been lost prior to analysis. Such losses are unlikely to be random, particularly given the strong links between health, employment status, and study participation.

The discussion includes some consideration of representativeness (lines 199–205), but there is little consideration of non-response related to poor health, socioeconomic disadvantage, or labour market detachment. These factors may have important implications for both external validity and internal validity.

Furthermore, no survey weights or other adjustments appear to have been applied. In the absence of formal assessment of representativeness or weighting procedures, claims that the analytical sample is representative should be removed or substantially qualified.

The authors should provide:

• Characteristics of included versus excluded participants where possible.

• Discussion of the potential for selection bias arising from non-response and attrition.

• Justification for any claims regarding representativeness.

4. Missing Data Handling

Missing data on items were handled by creating missing-value categories for covariates. While the proportion of missing data appears relatively very small, this approach has well-recognised limitations and may introduce bias into estimated associations. Dor example in the mutually adjusted models missingness was related to job satisfaction and expected income.

The economic inactivity data was ‘imputed’ using a single date following a set of rules established by the authors. This was the case for ‘469’ (line 94) participants – please could you state the number of times this was done. Also, this group seemed to be younger (less likely to be over 60). Using such an approach treats the value as a known, rather than an informed guesstimate. If used, could you call it ‘rule-based single imputation’ rather than imputed which is slightly misleading and outline the limitations.

Given the richness of the dataset, multiple imputation would appear both feasible and preferable for dealing with item non-response and attrition (to the sample of interest). Ideally, the analysis should be conducted with multiply imputed data or a sensitivity analysis comparing complete-case, missing-indicator, and multiple-imputation approaches should be presented to assess the robustness of findings. At minimum, the authors should justify their chosen approach and discuss its limitations further.

Additional Comments

5. Choice of Outcome Category

Line 187 indicates that the study used as justification for the outcome category - economic inactivity of 12 months or longer before SPa as similar to other studies. Reference Shaw et al. (2025) – this studies’ time period was very likely determined by interview timing during the COVID-19 pandemic and appears to be a pragmatic rather than scientifically motivated decision. The authors should provide a stronger justification for this choice.

Also, Line 195 the authors say, ‘the outcome that we chose was both more objective than self-reported retirement status, and a better measure of long-term impact on personal productivity’. How do they know this? Did they conduct a sensitivity analysis?

6. Mutually adjusted Modelling Strategy

Several aspects of the mutually adjusted analyses require clarification:

• Why was educational attainment excluded from the mutually adjusted model? It was a determinant for both men and women?

• Marital status appears to have been excluded for men because it was not statistically significant in descriptive analyses. However, marital status is a well-established determinant of retirement behaviour, caregiving responsibilities, health trajectories, and labour market participation. Variable inclusion in multivariable models should generally also be informed by theory and prior evidence rather than solely by statistical significance in univariable analyses. Consider retaining marital status for both men and women.

7. Potential Age-Stratified Analyses

Determinants of economic inactivity are likely to differ substantially across age bands within the “older worker” category. Some consideration should be given to motivations for inactivity nearer SPa (noted that the authors remove everyone 2 years prior to SPa) may differ markedly from those occurring earlier in later working life. Perhaps, sample permitting the manuscript would benefit from further exploration of heterogeneity by splitting the age group (at least as a sensitivity analysis) by age first left. Stratified analyses by age could strengthen the findings and improve interpretability.

8. Data Availability Statement

The manuscript states:

"The datasets used for this analysis are available on reasonable request from the MRC Lifecourse Epidemiology Centre by contacting the corresponding author."

This statement does not comply with PLOS ONE data availability requirements.

9. Writing and Presentation

The manuscript is generally understandable and reasonably well organised. However, there are several instances of repetitive phrasing, missing words, and minor grammatical issues. A careful editorial review would improve readability.

10. Also:

- line 44: ‘was first onset’ –needs clarifying - over the age of 50?

- line 64: ‘ Analysis was based on participants who, at the time of recruitment, were in a paid job…’ the study will possibly not include those temporarily LT sick at the time of interview, nor those, especially women coming back into the workplace. Should be mentioned as a limitation.

- Do you have information on the types of pensions participants had i.e. those with a Defined Benefit pension, and the type of work they did (e.g Standard Occupational Classification), public or private sector which are determinants of leaving the workforce?

Overall Assessment

This study addresses an important topic and utilises a valuable longitudinal dataset. However, the manuscript currently lacks sufficient engagement with the existing literature, does not adequately address the heterogeneity of economic inactivity, and raises concerns regarding representativeness, attrition, and missing data handling. Addressing these issues would substantially strengthen the scientific contribution and improve confidence in the reported findings.

Reviewer #3: 1. Sex-stratified analyses are insufficiently justified. Results are presented separately for men and women, but the manuscript does not state whether formal sex-by-exposure interaction terms were tested before stratification. The authors should either report interaction tests or justify sex stratification as an a priori analytic decision.

2 Potential selection and attrition bias. The analytic sample excludes participants not in paid work at baseline and those without the first two follow-up questionnaires, which may create a selected healthier or more stable working population. The authors should discuss how this may bias the observed associations.

3. Data-driven model selection. The multivariable model appears to include variables based on age-adjusted statistical significance or confidence interval criteria. This approach may omit important confounders and overemphasize chance findings; a theory-based confounder selection strategy would be preferable.

4 Unclear handling of time-varying exposures. Several predictors vary over time, but the manuscript uses the most recent known value at the start of each interval. This may misclassify exposure status during follow-up and should be more clearly justified.

5. Outcome timing may be imprecise. Extended labour market exit is defined as being continuously out of paid work for at least 12 months, but annual questionnaires and imputed dates may limit precision in identifying onset. The authors should clarify how timing uncertainty was handled.

6. Limited discussion of missing data. Missing values are included as categories in some analyses,but no clear multiple imputation or sensitivity analysis strategy is described. This may bias estimates if missingness is related to employment exit or key exposures.

7. Risk of multicollinearity and overadjustment. Financial difficulty, employment status,job insecurity, and work-related factors are likely correlated, and some may lie on causal pathways. The authors should clarify which variables are confounders versus mediators.

8. Interpretation may be too causal. The conclusions emphasize financial and employment-related drivers of exit, but the observational design limits causal inference.The wording should be more cautious.

9Sparse data in some subgroups. Some sex-specific estimates have wide confidence intervals, suggesting limited precision. These findings should be interpreted cautiously.

10. Proportional hazards or model assumptions are not discussed. The authors should state whether assumptions of the discrete-time Cox approach were checked.

11. Results are somewhat fragmented. Age-adjusted and mutually adjusted findings are spread across tables, making interpretation difficult. A clearer presentation of the main model would improve readability.

**********

what does this mean?). If published, this will include your full peer review and any attached files.

If you choose “no”, your identity will remain anonymous but your review may still be made public.

**Do you want your identity to be public for this peer review?**  For information about this choice, including consent withdrawal, please see our Privacy Policy

Reviewer #1: **Yes:**  Babak Eshrati

Reviewer #2: No

Reviewer #3: **Yes:**  AliAkbar Haghdoost

**********

[NOTE: If reviewer comments were submitted as an attachment file, they will be attached to this email and accessible via the submission site. Please log into your account, locate the manuscript record, and check for the action link "View Attachments". If this link does not appear, there are no attachment files.]

To ensure your figures meet our technical requirements, please review our figure guidelines: https://journals.plos.org/plosone/s/figures

You may also use PLOS’s free figure tool, NAAS, to help you prepare publication quality figures: https://journals.plos.org/plosone/s/figures#loc-tools-for-figure-preparation.

NAAS will assess whether your figures meet our technical requirements by comparing each figure against our figure specifications.

# Author comment: Author response to Decision Letter 1

**DOI:** 10.1371/journal.pone.0359865.r002

8 Sep 2026

Responses to comments raised by the editor and the reviewers are available in the attached file "Response to reviewers"

Attachment Submitted filename: Response to reviewers - Submit.docx

# Peer review: Decision Letter 1

**DOI:** 10.1371/journal.pone.0359865.r003

**Authors:** Hamid Reza Baradaran

20 Sep 2026

Associations of sociodemographic, health, and work-related factors with early long-term economic inactivity in older workers: findings from the Health and Employment After Fifty study

PONE-D-26-22418R1

Dear Dr. D'Angelo,

We’re pleased to inform you that your manuscript has been judged scientifically suitable for publication and will be formally accepted for publication once it meets all outstanding technical requirements.

Within one week, you’ll receive an e-mail detailing the required amendments. When these have been addressed, you’ll receive a formal acceptance letter and your manuscript will be scheduled for publication.

An invoice will be generated when your article is formally accepted. Please note, if your institution has a publishing partnership with PLOS and your article meets the relevant criteria, all or part of your publication costs will be covered. Please make sure your user information is up-to-date by logging into Editorial Manager at Editorial Manager® and clicking the ‘Update My Information' link at the top of the page. For questions related to billing, please contact billing support.

If your institution or institutions have a press office, please notify them about your upcoming paper to help maximize its impact. If they’ll be preparing press materials, please inform our press team as soon as possible -- no later than 48 hours after receiving the formal acceptance. Your manuscript will remain under strict press embargo until 2 pm Eastern Time on the date of publication. For more information, please contact onepress@plos.org.

Kind regards,

Hamid Reza Baradaran, M.D., Ph.D.,

Academic Editor

PLOS One

Additional Editor Comments (optional):

Reviewers' comments:

Reviewer's Responses to Questions

**Comments to the Author**

Reviewer #3: All comments have been addressed

**********

2. Is the manuscript technically sound, and do the data support the conclusions?

Reviewer #3: Yes

**********

3. Has the statistical analysis been performed appropriately and rigorously?

Reviewer #3: Yes

**********

4. Have the authors made all data underlying the findings in their manuscript fully available?

The PLOS Data policy

Reviewer #3: Yes

**********

5. Is the manuscript presented in an intelligible fashion and written in standard English?

Reviewer #3: Yes

**********

Reviewer #3: The authors have addressed my previous comments thoroughly and satisfactorily. I am satisfied with the revisions and have no further major concerns.

**********

what does this mean?). If published, this will include your full peer review and any attached files.

If you choose “no”, your identity will remain anonymous but your review may still be made public.

**Do you want your identity to be public for this peer review?**  For information about this choice, including consent withdrawal, please see our Privacy Policy

Reviewer #3: **Yes:**  AliAkbar Haghdoost

**********

# Editor report: Acceptance letter

**DOI:** 10.1371/journal.pone.0359865.r004

**Authors:** Hamid Reza Baradaran

PONE-D-26-22418R1

PLOS One

Dear Dr. D'Angelo,

I'm pleased to inform you that your manuscript has been deemed suitable for publication in PLOS One. Congratulations! Your manuscript is now being handed over to our production team.

At this stage, our production department will prepare your paper for publication. This includes ensuring the following:

* All references, tables, and figures are properly cited

* All relevant supporting information is included in the manuscript submission,

* There are no issues that prevent the paper from being properly typeset

You will receive further instructions from the production team, including instructions on how to review your proof when it is ready. Please keep in mind that we are working through a large volume of accepted articles, so please give us a few days to review your paper and let you know the next and final steps.

Lastly, if your institution or institutions have a press office, please let them know about your upcoming paper now to help maximize its impact. If they'll be preparing press materials, please inform our press team within the next 48 hours. Your manuscript will remain under strict press embargo until 2 pm Eastern Time on the date of publication. For more information, please contact onepress@plos.org.

You will receive an invoice from PLOS for your publication fee after your manuscript has reached the completed accept phase. If you receive an email requesting payment before acceptance or for any other service, this may be a phishing scheme. Learn how to identify phishing emails and protect your accounts at https://explore.plos.org/phishing.

If we can help with anything else, please email us at customercare@plos.org.

Thank you for submitting your work to PLOS One and supporting open access.

Kind regards,

PLOS One Editorial Office Staff

on behalf of

Professor Hamid Reza Baradaran

Academic Editor

PLOS One
